# Cardiorespiratory Fitness Decline After Short-Term Experimental Bed Rest in Older Adults and Ageing-Focused Cohorts Aged 55–65 Years: A Systematic Review

**DOI:** 10.3390/healthcare14203459

**Published:** 2026-10-15

**Authors:** Gustavo Vega-Fernández, Juan José Valenzuela-Fuenzalida, Yasna Jeria-Pizarro, Gustavo Oyanedel-Amaro, Mathias Orellana-Donoso, Gloria Cifuentes-Suazo, Juan Jose Cabezas-Salgado, Christopher Blackwood-Espinoza, José E. León-Rojas, Juan Sanchis-Gimeno, Rodrigo Cañas-Jamett, Nicolás Vidal-Seguel, Alejandro Bruna-Mejías

**Affiliations:** 1 Laboratory of Epidemiology and Morphological Sciences, Instituto de Biología, Pontificia Universidad Católica de Valparaíso, Valparaíso 2373223, Chile; gustavo.vega@pucv.cl; 2 Departamento de Morfología, Facultad de Medicina, Universidad Andrés Bello, Viña del Mar 2520000, Chile; juan.kine.2015@gmail.com (J.J.V.-F.); alejandro.bruna@unab.cl (A.B.-M.); 3 Departamento de Ciencias Químicas y Biológicas, Facultad de Ciencias de la Salud, Universidad Bernardo O’Higgins, Santiago 8370993, Chile; 4 Facultad de Ciencias de la Salud, Universidad Autónoma de Chile, Santiago 8910060, Chile; yasna.jeria@gmail.com; 5 Faculty of Health and Social Sciences, University of the Americas, Santiago 8370040, Chile; g.oyanedelamaro@gmail.com; 6 Escuela de Medicina, Universidad Finis Terrae, Santiago 7501015, Chile; mathor94@gmail.com; 7 Facultad de Medicina y Ciencia, Universidad San Sebastián, Lota 2465, Santiago 7510157, Chile; 8 Carrera de Odontología, Facultad de Medicina, Universidad Católica de la Santísima Concepción, Av. Alonso de Ribera 2850, Concepción 4090541, Chile; gbcifuentess@gmail.com; 9 Facultad de Medicina, Universidad Católica del Maule, Talca 3460000, Chile; jcabezas@ucm.cl; 10 Escuela de Ciencias de la Salud, Universidad Viña del Mar, Viña del Mar 2520000, Chile; christopher.blackwood@docente.uvm.cl; 11 Cerebro, Emoción y Conducta (CEC) Research Group, Escuela de Medicina, Universidad de las Américas (UDLA), Quito 170521, Ecuador; 12 GIAVAL Research Group, Faculty of Medicine, University of Valencia, 46001 Valencia, Spain; juan.sanchis@uv.es; 13 Physiology Laboratory, Department of Biological Sciences, Faculty of Life Sciences, Universidad Andrés Bello, Viña del Mar 2520000, Chile; rcanas@unab.cl; 14 Departamento de Ciencias Básicas, Facultad de Medicina, Universidad de La Frontera, Temuco 4811230, Chile; nicolasenrique.vidal@ufrontera.cl; 15 Departamento de Ciencias y Geografía, Facultad de Ciencias Naturales y Exactas, Universidad de Playa Ancha, Valparaíso 2360072, Chile

**Keywords:** bed rest, head-down tilt bed rest, cardiorespiratory fitness, oxygen uptake, older adults

## Abstract

**Background/Objectives**: Short-term bed rest may reduce cardiorespiratory capacity for mobility and rehabilitation, but gas-exchange-assessed fitness has not been synthesised in older adults. We characterised immediate ${\dot{V}O}_{2}$peak/${\dot{V}O}_{2}$max change, recovery, and countermeasures. **Methods**: This PROSPERO-registered review (CRD420261431685) searched seven databases and two trial registries through 24 May 2026, with Embase and SPORTDiscus through 26 May. Eligible studies imposed 5–14 days of horizontal bed rest (HBR) or 6° head-down tilt bed rest (HDBR) in cohorts with mean age ≥ 60 years or ageing-focused programmes enrolling adults aged 55–65 years. Reports sharing participants and protocol formed one cohort/protocol unit. Body-mass-normalised ${\dot{V}O}_{2}$ was primary; absolute ${\dot{V}O}_{2}$ was separate. Risk of bias was assessed with RoB 2 or the revised JBI tool, and certainty with GRADE. Meta-analysis was not undertaken because three independent, comparable effects were unavailable. **Results**: Of 1964 records, 15 reports formed six units representing four to six independent participant groups. Across four HBR units (*n* = 9–19), ${\dot{V}O}_{2}$peak/${\dot{V}O}_{2}$max declined by 4.9–15.3%; two percentages were directly reported, one was calculated from a reported change, and one from unit-adjudicated means (low certainty for direction; very low for magnitude). In one HDBR trial, changes were −13.4% ± 12.5% SD in controls (*n* = 9) and −3.5% ± 5.2% SD with multimodal exercise (*n* = 10). Single leucine, approximately 2000 steps/day, and whey comparisons provided insufficient evidence of ${\dot{V}O}_{2}$ preservation. Recovery evidence from three programmes was heterogeneous and very uncertain; only one small HDBR cohort had approximately four-week follow-up. **Conclusions**: ${\dot{V}O}_{2}$peak/${\dot{V}O}_{2}$max consistently declined after short-term HBR, but a precise decline, duration–response relationship, specific countermeasure prescription, and universal recovery time are not established. Minimising avoidable immobility is a plausible clinical implication, not a directly tested intervention. No external funding.

## 1. Introduction

Cardiorespiratory fitness (CRF), commonly expressed as peak or maximal oxygen uptake (${\dot{V}O}_{2}$peak/${\dot{V}O}_{2}$max), integrates cardiovascular, pulmonary, haematological, and skeletal-muscle function and is increasingly regarded as a clinically meaningful vital sign [1]. Because it quantifies the capacity to sustain whole-body exercise, lower CRF can constrain exercise tolerance, functional independence, mobility, and rehabilitation capacity. Aerobic capacity declines with age, and even healthy adults experience an accelerated longitudinal reduction in peak oxygen uptake later in life [2]. A brief period of severe inactivity may therefore be particularly consequential when baseline fitness is already limited.

Bed rest is a controlled model of inactivity and unloading. Classical studies established rapid losses in cardiovascular performance and work capacity during prolonged bed rest [3,4]. A previous meta-analysis of predominantly young adults synthesised ${\dot{V}O}_{2}$max decline across bed-rest durations of 1–90 days [5], whereas reviews focused on older participants have primarily addressed muscle mass, strength, and function rather than CRF [6]. No previous synthesis has jointly focused on gas-exchange-assessed CRF after 5–14 days of experimental bed rest in older adults, later-middle-aged ageing models, recovery trajectories, and countermeasure findings.

The evidence is also structurally fragmented. A single bed-rest campaign may generate multiple primary, mechanistic, protocol, and conference reports; counting these reports as independent studies can duplicate participants or controls and exaggerate apparent sample size, precision, and consistency. In addition, conventional HBR primarily models inactivity and unloading, whereas 6° HDBR also imposes an anti-orthostatic cephalad fluid shift. These models were therefore not assumed to be quantitatively equivalent.

The primary objective was to characterise the direction and observed magnitude of the immediate change in ${\dot{V}O}_{2}$peak/${\dot{V}O}_{2}$max after 5–14 days of experimental HBR or HDBR in older adults and prespecified ageing-focused cohorts. Secondary objectives were to describe later recovery after remobilisation or structured rehabilitation and to evaluate exercise, mobility, and nutritional countermeasure findings.

## 2. Materials and Methods

### 2.1. Protocol, Registration, and Reporting

The protocol was prospectively registered in PROSPERO (CRD420261431685). Reporting followed PRISMA 2020 and PRISMA-S [7,8]; search development was guided by PRESS principles [9], and synthesis reporting followed SWiM [10]. The review question, population pathways, exposure window, eligibility criteria, and primary outcome were unchanged. Meta-analysis was conditional on the availability of at least three independent, sufficiently comparable cohort-level effects; because this feasibility condition was not met, the prespecified structured-synthesis pathway was implemented. Administrative clarifications to the title, search dates, risk-of-bias tools, outcome reporting, and completed synthesis are documented in Supplementary S8.

### 2.2. Information Sources and Search Strategy

MEDLINE/PubMed, Scopus, Web of Science Core Collection, CENTRAL, and CINAHL were searched from inception to 24 May 2026; Embase and SPORTDiscus were searched to 26 May 2026. ClinicalTrials.gov and the WHO International Clinical Trials Registry Platform were searched on 24 May 2026 for registrations and linked reports. Registry records were used for registration/report linkage and did not contribute separate bibliographic screening records. No language or publication-date restrictions were applied. Database-specific strategies combined controlled vocabulary and free-text terms for bed rest or HDBR with ${\dot{V}O}_{2}$peak, ${\dot{V}O}_{2}$max, oxygen uptake, aerobic capacity, CRF, and exercise testing. Backward and forward citation searching identified no additional unique screening records after matching against the deduplicated library (*n* = 0), but supported companion-report linkage and cohort mapping. Full strategies are provided in Supplementary S1.

### 2.3. Eligibility Criteria, Outcomes, and Time-Point Hierarchy

For this review, short-term bed rest was operationalised a priori as 5–14 days to focus on acute deconditioning within an approximately one- to two-week interval and to avoid combining this evidence with longer campaigns carrying substantially different cumulative exposure. Eligible populations met either of two prespecified pathways: (1) a cohort mean age ≥ 60 years; or (2) an explicitly ageing-focused experimental programme enrolling all participants within the 55–65-year age range. The second pathway captured studies prospectively designed to examine later-middle-age or ageing physiology and did not redefine all adults aged 55–59 years as older adults. Mixed-age studies were eligible only when data for an eligible subgroup were separately extractable.

The primary outcome was body-mass-normalised peak or maximal oxygen uptake (mL·kg^−1^·min^−1^) assessed during an incremental exercise test using respiratory gas-exchange analysis. An equivalent measure was accepted when the report described the highest or maximal gas-exchange oxygen-uptake value from an eligible peak or maximal exercise protocol, irrespective of whether the authors labelled it ${\dot{V}O}_{2}$peak or ${\dot{V}O}_{2}$max. Values estimated solely from workload-based predictive equations were excluded. Absolute ${\dot{V}O}_{2}$ (L·min^−1^) was secondary and was analysed separately because body mass may change during bed rest. According to the prespecified time-point hierarchy, the closest valid baseline was the last technically valid pre-bed-rest assessment; the earliest post-bed-rest assessment was the first technically valid test after the exposure, including recovery day 1 or 2 when no in-bed maximal test was available.

Recovery was defined as the trajectory from the earliest post-bed-rest assessment to a later post-remobilisation or post-rehabilitation assessment, while also retaining comparison with the pre-bed-rest baseline. Natural return to usual activity and structured rehabilitation were described separately. Randomised and non-randomised countermeasure studies and uncontrolled before–after protocols were eligible. Step reduction without bed rest, limb immobilisation, dry immersion, spaceflight, uncontrolled hospital inactivity, workload-only estimates of ${\dot{V}O}_{2}$max, and exposures outside the 5–14-day window were excluded.

### 2.4. Study Selection, Cohort Linkage, and Data Extraction

Records were managed in Zotero version 9.0.4. Automated and manual deduplication, followed by a residual duplicate audit, yielded 1006 unique records. Title/abstract screening and full-text eligibility assessment were completed in duplicate by A.B.-M. and J.J.V.-F. using predefined criteria; disagreements were resolved by G.O.-A. Agreement for title/abstract screening was substantial (Cohen’s κ = 0.80). One principal PRISMA reason was assigned to each full-text exclusion.

A cohort/protocol unit was defined as a distinct participant group exposed to a specific bed-rest programme. Publications, protocols, registry records, conference abstracts, and secondary analyses were linked using trial registration, centre, investigators, recruitment and sample characteristics, exposure model and duration, intervention arms, diet, assessment schedule, and cross-citations. Reports from the same participants were not counted independently; shared controls were used once; and distinct cohorts or protocols were separated when independently identifiable. BROA/CAIS reports were treated as one HDBR unit, the leucine, whey, and approximately 2000-step reports as one UTMB unit with potentially shared controls, and the earlier metabolic report from the Coker programme as an associated report. Independence remained unresolved for the Coker–Kortebein and Pišot–Zuccarelli pairs.

A piloted form captured population, exposure, co-interventions, CPET protocol, assessment timing, ${\dot{V}O}_{2}$ values, dispersion, recovery, and secondary outcomes. Data extraction and study coding were performed by G.O.-A., R.C.-J., and G.C.-S. using standardised forms. Directly reported values appeared explicitly in a report; calculated values were arithmetic transformations of reported data; derived values reconstructed an approximate post value or change from another reported statistic; figure-derived values were extracted from a graph using a documented procedure; unit-adjudicated values retained a published value whose stated unit was internally inconsistent and was resolved through source triangulation; and abstract-only values lacked a complete full report. Directly reported values were prioritised. SD and SEM were retained in their reported form and were not treated as interchangeable.

### 2.5. Risk of Bias, Certainty, and Synthesis

Risk of bias was assessed at outcome or finding level by M.O.-D., A.B.-M., and J.J.C.-S. Randomised countermeasure results were assessed with RoB 2, whereas pre–post-exposure findings, recovery trajectories, and non-randomised comparisons were assessed with the revised JBI quasi-experimental tool [11,12]. Final judgements were reconciled before synthesis. Certainty was rated with GRADE for seven prespecified findings, separating certainty about the direction of the immediate HBR effect from certainty about its magnitude [13].

Meta-analysis required at least three independent effects representing a sufficiently common bed-rest model, outcome metric and unit, assessment time, comparator, data provenance, and usable measure of uncertainty. This condition was not met because of unresolved cohort dependence, potentially shared controls, differences between HBR and HDBR, variation in relative versus absolute ${\dot{V}O}_{2}$ and post-exposure timing, and reliance in some findings on calculated, derived, unit-adjudicated, or incompletely reported data. HBR and HDBR were therefore synthesised separately using structured quantitative and narrative methods, with emphasis on direction, observed range, timing, risk of bias, and certainty rather than statistical significance. No pooled effect, heterogeneity statistic, or small-study-effect test was calculated.

Quantitative data processing, descriptive calculations, inter-reviewer agreement analyses, and non-pooled visualisations were performed in R version 4.5.2 within RStudio version 2026.04.0+526. The metafor package version 5.0-1 was used for study-level calculations and the non-pooled evidence display; no summary effect was estimated. Expanded methods are provided in Supplementary S2–S7. Ethics approval was not required because only published aggregate data were used.

## 3. Results

### 3.1. Study Selection and Cohort/Protocol Architecture

The searches identified 1964 records; after removal of 958 duplicates, 1006 unique records were screened. Thirty-one reports were sought, eight were not retrieved, and 23 were assessed in full text. The eight non-retrieved reports (25.8% of reports sought) comprised six publications from 1986 to 2000 without a reported DOI and two FASEB conference abstracts from 2016 and 2017. Their eligibility and result direction could not be verified, so their potential impact was treated as uncertain in the assessment of missing results. The retained retrieval archive does not allow verification of whether author contact was attempted for each report; no such contact is therefore claimed. Eight retrieved reports were excluded, and 15 reports were retained for synthesis or cohort mapping (Figure 1; Supplementary S2).

Five units used HBR and one used HDBR. The 15 reports did not represent 15 independent studies. At minimum, the evidence comprised four mutually distinct participant networks: BROA/CAIS, UTMB, the unresolved Coker–Kortebein pair, and the unresolved Pišot–Zuccarelli pair. If both unresolved pairs were independent, the maximum was six participant groups. Analytical sample sizes for individual ${\dot{V}O}_{2}$ findings ranged from 9 to 19 participants. Seven reports arose from BROA/CAIS [14,15,16,17,18,19,20], three from the UTMB programme [21,22,23], two from the Coker programme [24,25], and one each from Kortebein [26], Pišot [27], and Zuccarelli [28]. The report linkage and unresolved relationships are shown in Figure 2. Table 1 summarises the characteristics, measurement methods, assessment timing, data provenance, and risk of bias of the six cohort/protocol units.

### 3.2. Immediate Changes After HBR and HDBR

${\dot{V}O}_{2}$peak/${\dot{V}O}_{2}$max declined after 7–14 days in each of the four principal HBR units (Figure 3), but the estimates differed in provenance and dispersion. In the UTMB alanine-control arm, the directly reported change was −1.2 ± 0.9 SEM mL·kg^−1^·min^−1^ from a baseline of 24.5 ± 1.9 SEM; the post-bed-rest mean of 23.3 and the 4.9% decline were calculated by the review [21]. Coker reported 21.5 ± 1.3 to 18.6 ± 1.0 SEM; the source table unit was internally incompatible with the values and was adjudicated as mL·kg^−1^·min^−1^, yielding a calculated 13.5% decline [24]. Kortebein directly reported a −12.2% ± 4.5% SEM change [26], and Pišot directly reported −15.3% ± 11.0% SD in older men [27]. The Zuccarelli abstract reported an approximately 5% decline (*p* = 0.02) without a complete post-bed-rest value or dispersion and was retained only as narrative support [28].

In the single HDBR control cohort, the directly reported peak ${\dot{V}O}_{2}$ change at recovery day 1 or 2 was −13.4% ± 12.5% SD (*n* = 9) [14]. Two control peak tests were invalid at this assessment, and some affected peak values were reconstructed using an internally validated personalised model; this was retained as a provenance and risk-of-bias caveat. The apparent ordering from a smaller decline after 7 days to larger declines after 10–14 days was descriptive only. Duration was inseparable from cohort, sex, baseline fitness, bed-rest model, CPET protocol, post-exposure timing, and data provenance, so no duration–response relationship was inferred.

### 3.3. Countermeasure Findings

One small randomised HDBR trial compared approximately 1 h/day of high-intensity interval and aerobic cycling plus upper- and lower-body resistance exercise with a control condition without an exercise countermeasure [14]. The source reported a group-by-time interaction for peak ${\dot{V}O}_{2}$ (*p* = 0.002). At recovery day 1 or 2, the directly reported percentage changes were −3.5% ± 5.2% SD in the exercise group (*n* = 10) and −13.4% ± 12.5% SD in controls (*n* = 9). From these arm-level summaries, the review-calculated unadjusted difference in percentage-point change was 9.9 percentage points favouring exercise (95% CI −0.02 to 19.82; Welch t method). This single-time-point contrast is not equivalent to the source repeated-measures interaction and is not an adjusted treatment effect.

Three additional single comparisons arose from the same UTMB programme and could include shared controls. In the leucine comparison, isoenergetic meals supplemented with leucine 0.06 g·kg^−1^·meal^−1^ (*n* = 10) were compared with alanine 0.06 g·kg^−1^·meal^−1^ (*n* = 10) during 7-day HBR; the reported changes were −3.4 ± 1.1 and −1.2 ± 0.9 SEM mL·kg^−1^·min^−1^, respectively [21]. The STEP comparison delivered 2018 ± 4 steps/day, approximately 22 ± 1 min/day, during HBR; ${\dot{V}O}_{2}$ data were available for six intervention and ten control participants, but marked baseline imbalance and one missing intervention result precluded a reliable benefit estimate [22]. In the non-randomised WHEY comparison, a whey-isolate-enriched diet provided approximately 70% of dietary protein from whey during 7-day HBR, whereas the MIXED diet used varied plant and animal sources; each group included ten participants. Large baseline ${\dot{V}O}_{2}$ imbalance and unclear allocation limited interpretation [23]. Together, these single comparisons were insufficient to determine whether leucine, approximately 2000 steps/day, or a whey-enriched diet preserved ${\dot{V}O}_{2}$; they do not demonstrate equivalence or absence of effect.

### 3.4. Recovery Findings

Recovery was evaluated in three programmes with different follow-up schedules and rehabilitation exposure. After 7-day HBR and five days of structured inpatient rehabilitation, the UTMB alanine-control mean remained approximately 4.9% below baseline, although it was no longer statistically different from baseline [21]. In older men after 14-day HBR and 14 days of protocolised rehabilitation, the directly reported change remained −9.3% ± 13.4% SD relative to baseline [27]. In the HDBR control cohort, complete-case absolute peak ${\dot{V}O}_{2}$ means were 2.00 ± 0.66 L·min^−1^ at baseline (*n* = 6), 1.79 ± 0.58 at recovery day 1 or 2, 1.89 ± 0.56 at day 6, and 2.06 ± 0.58 at approximately four weeks [14]. Longitudinal models used all available observations, but post-discharge activity was not monitored, and the four-week complete-case sample was small. Recovery timing was therefore not considered fixed or generalisable.

### 3.5. Risk of Bias

For immediate exposure findings, Kortebein and Pišot were judged to be low concern; Hedge, the UTMB control, and Coker had some concerns; and the Zuccarelli abstract had insufficient information. All three recovery findings had some concerns. The multimodal-exercise and leucine comparisons had some concerns, the STEP comparison was at high risk, and the WHEY comparison had high concern. Judgements reflected missing or reconstructed measurements, incomplete maximality reporting, unit inconsistency, small samples, baseline imbalance, unclear allocation, and analysis flexibility as applicable (Supplementary S6).

### 3.6. Certainty of Evidence

GRADE certainty was low for the direction of the immediate HBR effect and very low for its magnitude, any duration pattern, the HDBR finding, countermeasures, and recovery (Table 2). The evidence therefore supports a cautious statement about the consistency of observed decline but not a precise expected percentage, a causal duration–response relation, a definitive countermeasure prescription, or a universal recovery time.

## 4. Discussion

### 4.1. Principal Findings and Relation to Previous Evidence

${\dot{V}O}_{2}$peak/${\dot{V}O}_{2}$max consistently declined across the four principal HBR cohort/protocol units. The observed 4.9–15.3% range is not a pooled effect or an expected individual decline; it arose from small heterogeneous samples and mixed data provenance. Accordingly, certainty was low for the direction and very low for the magnitude.

The direction is concordant with the earlier Ried-Larsen synthesis, which included 80 studies and 949 participants, more than 90% men, with a median age of 24.5 years and bed-rest durations of 1–90 days [5]. That review described a broadly linear decline in ${\dot{V}O}_{2}$max across duration. The present review addressed a different and much smaller evidence base focused on older or ageing-model cohorts. It did not include a direct pooled age comparison, and therefore cannot establish that older adults experience a larger decline than younger adults, even though individual included reports have suggested this possibility.

The apparent ordering of the current estimates by 7, 10, and 14 days cannot establish a dose–response relation. Duration was confounded with the participating cohort, sex distribution, baseline fitness, HBR versus HDBR, CPET protocol, timing of post-bed-rest testing, and whether an estimate was directly reported, calculated, or adjudicated.

### 4.2. HBR, HDBR, and Countermeasures

HBR and HDBR should not be quantitatively equated. Both impose severe inactivity and unloading, but HDBR additionally produces an anti-orthostatic cephalad fluid shift and a distinct haemodynamic context. Moreover, the first BROA peak ${\dot{V}O}_{2}$ assessment occurred on recovery day 1 or 2 rather than during HDBR. Directional concordance with HBR is therefore informative, but one HDBR cohort cannot establish quantitative equivalence between models. Changes in plasma volume, preload, stroke volume, maximal heart rate, peripheral oxygen extraction, and the timing or familiarisation of post-bed-rest testing could plausibly influence the observed ${\dot{V}O}_{2}$ response [3,4,15,18,20]. However, these variables were not assessed consistently across the principal ${\dot{V}O}_{2}$ reports, and the linked mechanistic or secondary reports do not permit attribution of the decline to a specific central or peripheral pathway. They are therefore considered plausible contributors rather than mechanisms demonstrated by this review.

The multimodal exercise trial showed a smaller early decline and a source-reported group-by-time interaction, supported by ventilatory-threshold and submaximal outcomes [14]. However, the intervention combined high-intensity interval cycling, aerobic cycling, and upper- and lower-body resistance exercise for approximately one hour daily under intensive supervision. One small trial cannot identify the effective component, optimal dose, safety profile, or a clinical prescription for hospitalised older adults. An arm-level adherence percentage linked specifically to the ${\dot{V}O}_{2}$ analysis was not available in the review dataset. Accordingly, this finding should be interpreted as evidence concerning the intensively supervised multimodal protocol evaluated in that trial rather than exercise in general.

The leucine, STEP, and WHEY findings are even less conclusive. Each was represented by one small comparison, and all arose from the same UTMB programme with potential control overlap. Leucine was compared with an isonitrogenous alanine control, the STEP intervention delivered approximately 2000 steps/day, and WHEY altered protein quality without increasing total energy intake. The available analyses were insufficient to determine whether any of these interventions preserved ${\dot{V}O}_{2}$; their imprecision, baseline imbalance, missing data, and allocation concerns also preclude conclusions of equivalence or no effect.

### 4.3. Recovery and Potential Modifiers

Recovery evidence was heterogeneous in both timing and exposure. The UTMB and Pišot programmes included structured rehabilitation, whereas most of the later BROA follow-up reflected return to usual living after the first supervised week. Residual numerical deficits after 5–14 days and the four-week HDBR trajectory therefore represent different recovery contexts. The four-week observation derives from one small cohort and should not be generalised as a universal recovery time.

Baseline fitness, sex, post-bed-rest activity, nutrition, and rehabilitation exposure may modify recovery, but the included evidence was too sparse to estimate interactions. Hedge reported substantial individual heterogeneity and exploratory relationships between baseline submaximal fitness and early change, whereas the peak ${\dot{V}O}_{2}$ association was not statistically significant [14]. Future studies should prespecify these potential modifiers and monitor activity after discharge or release from the laboratory.

### 4.4. Clinical Implications, Strengths, and Limitations

The consistent experimental decline makes avoidance of unnecessary immobility a clinically plausible implication, not a directly tested intervention effect. The participants were generally healthy or clinically stable, relatively active, intensively monitored volunteers. Hospitalised, frail, multimorbid, inflamed, medicated, or malnourished older patients may have different baseline capacity, catabolic exposure, exercise tolerance, and recovery. The direction of deconditioning may be clinically relevant, but the experimental percentage range and recovery timing are not directly transferable to those populations.

Strengths include prospective registration, broad searching, residual deduplication, duplicate study selection, explicit linkage of reports to cohort/protocol units, separation of HBR and HDBR, transparent data provenance, outcome-level risk-of-bias assessment, and finding-level GRADE. The decision not to pool followed a prespecified feasibility rule and avoided false precision from dependent or incompatible effects.

Limitations include the small and fragmented evidence base, unresolved Coker–Kortebein and Pišot–Zuccarelli relationships, inconsistent CPET protocols and post-exposure timing, limited representation of the oldest-old and frailty, male-only cohorts, and values requiring calculation or unit adjudication. Eight of 31 reports sought were not retrieved; because their eligibility and results could not be established, the direction of any resulting selection bias is unknown. Formal small-study-effect tests were not informative with so few potentially independent units.

### 4.5. Research Priorities

Future bed-rest studies should use prospective registration and persistent cohort identifiers so that linked publications and shared controls can be recognised. Reports should provide a standardised CPET protocol and maximality criteria, relative and absolute ${\dot{V}O}_{2}$, complete arm-level means and variance, reasons for missing tests, and prespecified common follow-up points. Countermeasure trials should report exercise or nutritional dose, adherence, adverse events, baseline-adjusted between-group effects, and potential effect modifiers including frailty, multimorbidity, sex, and baseline fitness.

## 5. Conclusions

${\dot{V}O}_{2}$peak/${\dot{V}O}_{2}$max consistently declined after 7–14 days of HBR across the principal cohort/protocol units, but low-certainty evidence limits causal inference. Current evidence does not establish a precise expected percentage decline or a reliable duration–response relationship. One small HDBR trial suggested that daily supervised multimodal exercise may attenuate deconditioning, but it does not establish a specific prescription. Single leucine, whey-diet, and approximately 2000-step comparisons were insufficient to determine ${\dot{V}O}_{2}$ preservation. Recovery may remain incomplete during the first one to two weeks; the observation that ${\dot{V}O}_{2}$ approached baseline by approximately four weeks derives from one small HDBR cohort and should not be generalised.

## Figures and Tables

**Figure 1:**
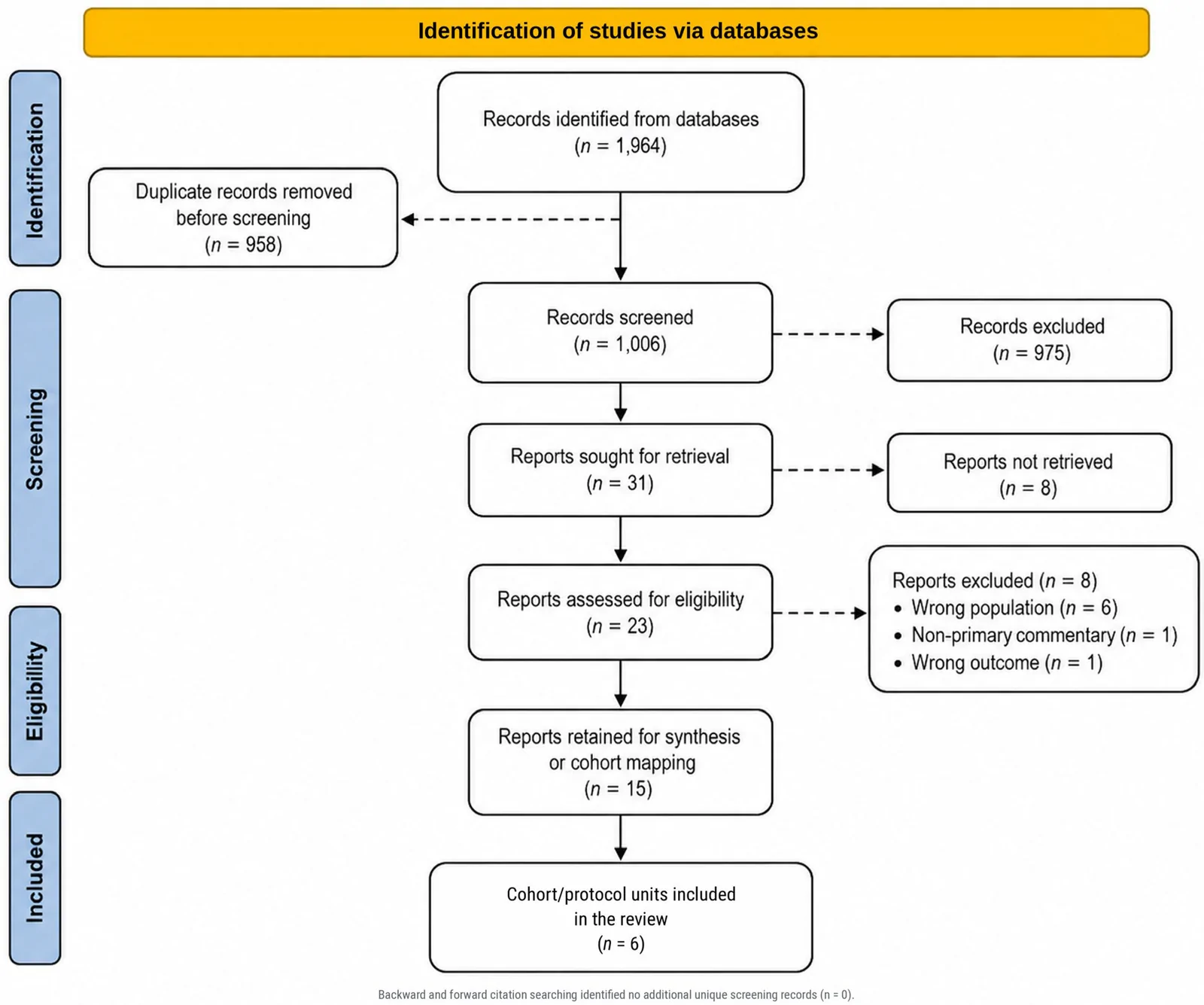
PRISMA 2020 flow diagram. Solid arrows show progression through identification, screening, retrieval, eligibility, and synthesis; dashed arrows show duplicate removal or exclusion. Citation searching identified no additional unique screening records (*n* = 0). Fifteen retained reports were linked to six cohort/protocol units. Trial registries supported registration/report linkage and did not contribute separate bibliographic screening records.

**Figure 2:**
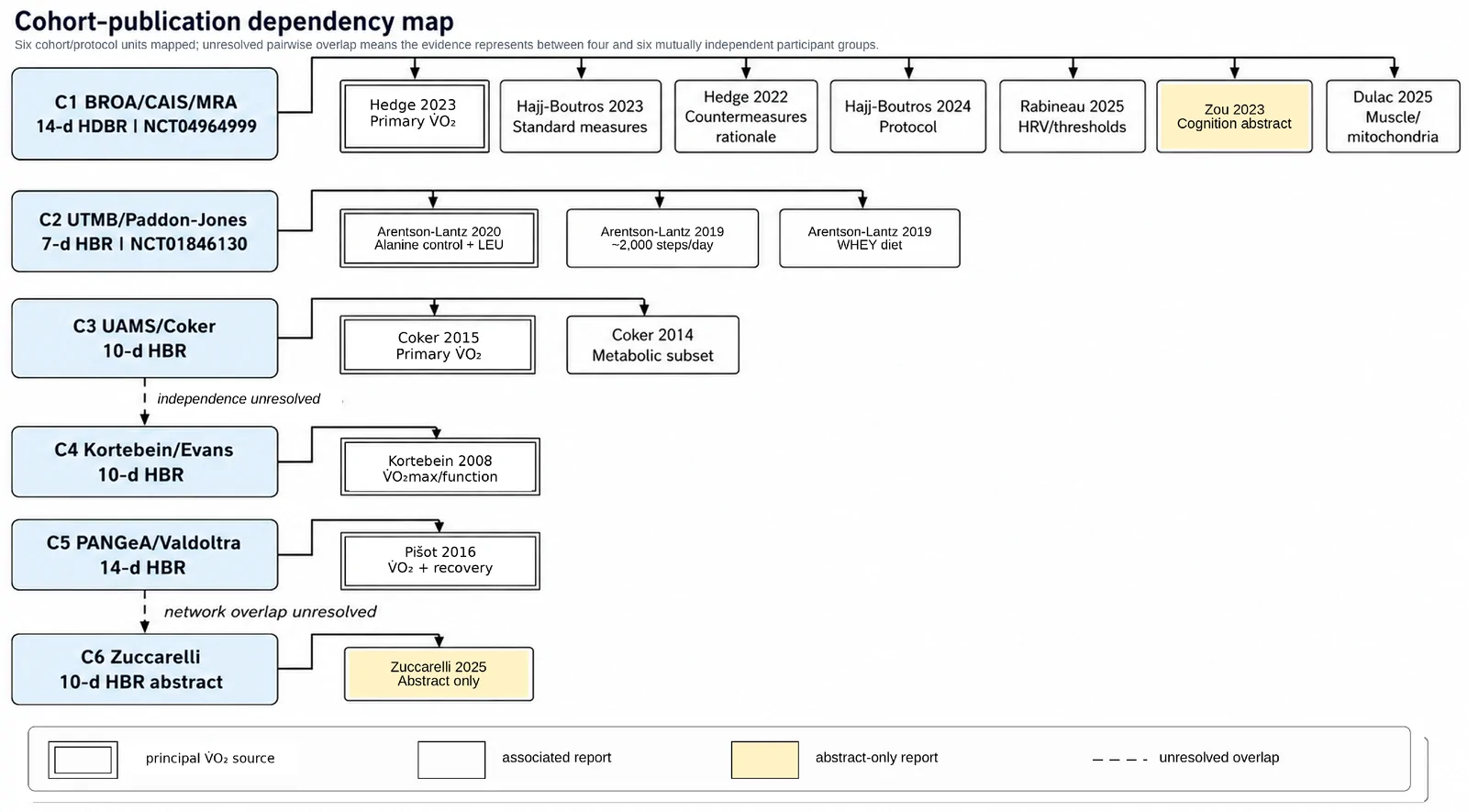
Cohort–publication dependency map. Large blue boxes represent the six cohort/protocol units. Double-bordered report boxes identify the principal ${\dot{V}O}_{2}$ source; single-bordered boxes identify associated reports; yellow boxes identify abstract-only reports; dashed links identify unresolved overlap. The six units represent between four and six mutually independent participant groups [14,15,16,17,18,19,20,21,22,23,24,25,26,27,28].

**Figure 3:**
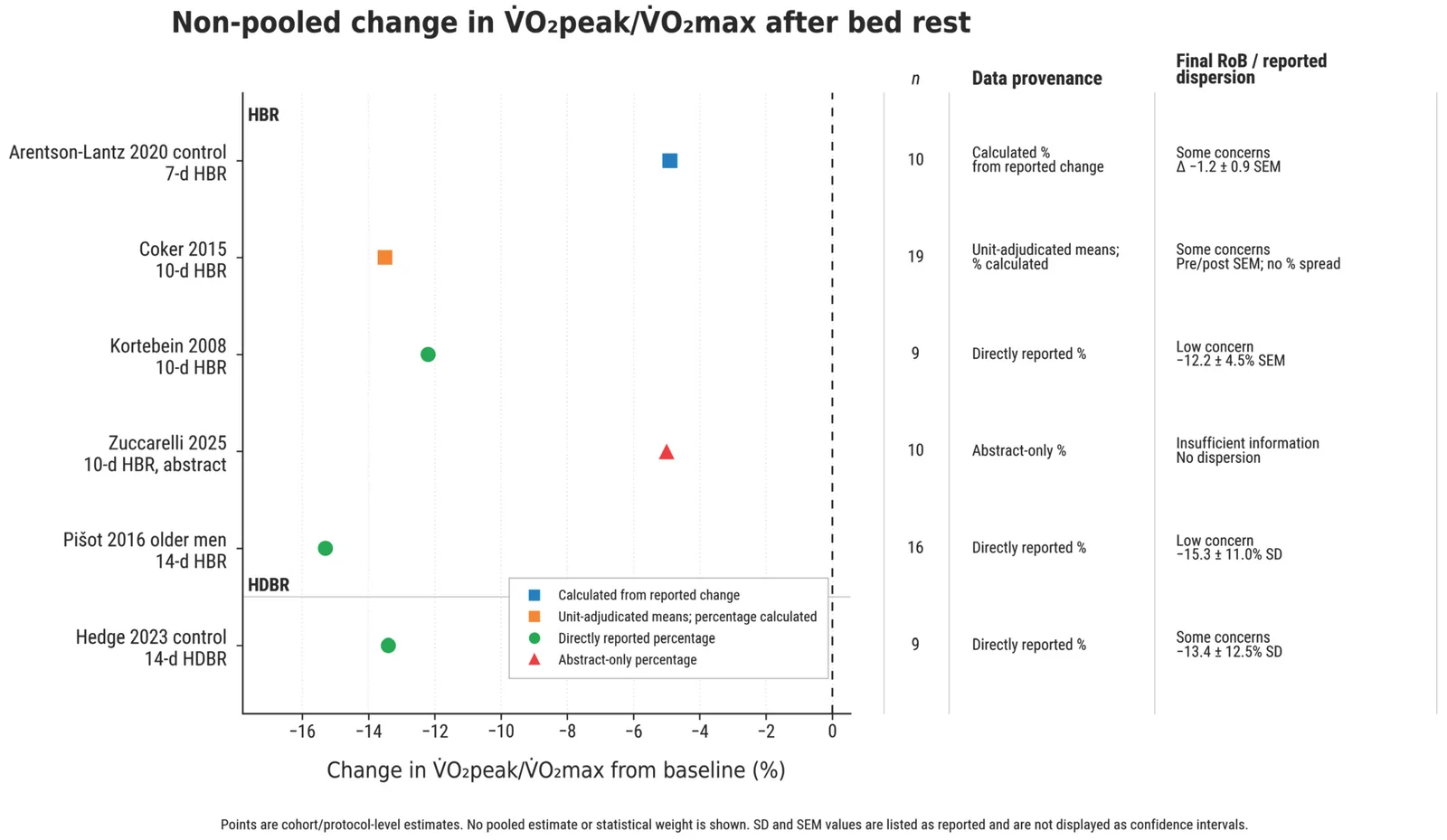
Non-pooled changes in ${\dot{V}O}_{2}$peak/${\dot{V}O}_{2}$max after short-term bed rest. Points show cohort/protocol-level percentage changes from baseline and marker shape indicates data provenance. The adjacent columns provide analytical sample size, final outcome-level risk of bias, and the SD or SEM as originally reported. Dispersion measures were not converted to confidence intervals and are therefore not plotted as horizontal intervals. HBR and HDBR are displayed separately; no pooled estimate, statistical weight, or summary diamond is shown [14,21,24,26,27,28].

**Table 1:** Characteristics, measurement methods, timing, provenance, and risk of bias of the cohort/protocol units.

Unit/Setting	Population and Analytical *n*	Model; Intervention/Comparator	CPET and ${\dot{V}O}_{2}$ Metric	Assessment Timing	Data Provenance	Final Outcome-Level RoB/Role
C1 BROA/CAIS/MRA [14,15,16,17,18,19,20] Canada	Healthy adults aged 55–65; 11 women/11 men; R + 1/2 *n* = 9 control and *n* = 10 exercise	6° HDBR; 14 d; ~1 h/day multimodal exercise vs. no exercise countermeasure/passive control	Incremental cycle test; breath-by-breath gas exchange; highest 20 s average; relative and absolute peak ${\dot{V}O}_{2}$	PRE; R + 1/2; R + 6; R + 4 weeks	Directly reported percentage changes; complete-case absolute means; some peak tests reconstructed with a validated model	Some concerns for immediate exposure and exercise; principal HDBR, countermeasure, and recovery unit
C2 UTMB/NCT01846130 [21,22,23] USA	Healthy adults aged 60–80; mixed sex; *n* = 10 principal control; LEU *n* = 10; STEP ${\dot{V}O}_{2}$ *n* = 6; WHEY/MIXED *n* = 10/10	Strict HBR; 7 d; alanine control vs. leucine; no-weight-bearing control vs. ~2000 steps/day; MIXED vs. whey-enriched diet	Graded cycle ergometry with metabolic cart; relative peak ${\dot{V}O}_{2}$	Baseline; post-BR; after 5 d inpatient rehabilitation	Direct changes with SEM; post means and percentages calculated; shared/overlapping controls	Some concerns for exposure/LEU; high risk for STEP; high concern for WHEY
C3 Coker programme [24,25] USA	Clinically stable adults aged 60–85; 14 women/5 men; *n* = 19	Strict HBR; 10 d; uncontrolled before–after	Cycle ergometry with respiratory gas analysis; relative peak ${\dot{V}O}_{2}$ in primary report; absolute ${\dot{V}O}_{2}$ in associated subset	Closest pre-BR assessment; immediate post-BR	Pre/post means reported with SEM; primary table unit adjudicated as mL·kg^−1^·min^−1^; percentage calculated	Some concerns; primary 10 d HBR unit with mandatory unit caveat
C4 Kortebein programme [26] USA	Healthy adults aged 62–75; mixed sex; ${\dot{V}O}_{2}$ *n* = 9	Strict HBR; 10 d; uncontrolled before–after	Electrically braked cycle; staged/incremental protocol to volitional fatigue or ACSM termination; relative ${\dot{V}O}_{2}$max	Pre-BR and after completion of HBR	Direct pre/post means and directly reported percentage change, all with SEM	Low concern; corroborative 10 d HBR unit; independence from C3 unresolved
C5 PANGeA/Valdoltra [27] Slovenia/Italy	Healthy men aged 55–65; *n* = 16	HBR; 14 d; older subgroup; structured rehabilitation during recovery	Incremental cycle ergometry; relative peak ${\dot{V}O}_{2}$	PRE; BR14; R + 14	Directly reported percentage changes with SD; absolute post values derived only for secondary display	Low concern for immediate exposure; some concerns for recovery; principal 14 d HBR unit
C6 Zuccarelli abstract [28] Italy/Slovenia	Healthy men; mean age 69; *n* = 10	HBR; 10 d; abstract-level protocol	Incremental exercise test; peak pulmonary ${\dot{V}O}_{2}$; methods incompletely reported	Baseline and POST10	Abstract-only approximate percentage; no complete post value or dispersion	Insufficient information; narrative only; relationship with C5 unresolved

Notes: A cohort/protocol unit is a distinct participant group exposed to a specific bed-rest programme. Participant totals were not summed across linked or potentially overlapping reports. PRE, pre-bed-rest; R, recovery; BR, bed rest; SD, standard deviation; SEM, standard error of the mean; HBR, horizontal bed rest; HDBR, head-down tilt bed rest; RoB, risk of bias. ACSM, American College of Sports Medicine; LEU, leucine-supplemented comparison; STEP, approximately 2000 steps/day intervention; WHEY, whey-isolate-enriched diet; MIXED, mixed-protein comparator.

**Table 2:** Finding-level summary of findings.

Critical Finding	Evidence Base	Structured Result	Certainty	Plain-Language Conclusion
Direction after 7–14 d HBR	Four principal cohort/protocol units	${\dot{V}O}_{2}$ consistently declined; observed changes 4.9–15.3% with mixed provenance	Low	Observed decline was consistent; low-certainty evidence suggests that short-term HBR may reduce CRF.
Magnitude and duration pattern	Same four HBR units; heterogeneous metrics, timing, and provenance	Two percentages were directly reported; one was calculated from a reported change and one from unit-adjudicated means; no pooled estimate	Very low	A precise expected decline and a reliable duration–response relationship are not established.
Immediate 14 d HDBR	One control cohort; *n* = 9	Directly reported −13.4% ± 12.5% SD at R + 1/2; some peak values reconstructed	Very low	HDBR was associated with early fitness loss, but the magnitude is very uncertain.
Multimodal exercise	One small randomised HDBR trial; *n* = 10 vs. 9	Source group-by-time *p* = 0.002; review-calculated unadjusted difference 9.9 percentage points (95% CI −0.02 to 19.82)	Very low	Exercise may attenuate decline, but one trial does not establish an exercise prescription.
Leucine or whey	One leucine comparison and one non-randomised whey comparison within one programme	Evidence from one leucine and one whey comparison was insufficient to determine ${\dot{V}O}_{2}$ preservation; shared controls and baseline imbalance limit inference	Very low	Evidence is insufficient to determine whether protein-based strategies preserve ${\dot{V}O}_{2}$.
Approximately 2000 steps/day	One very small randomised comparison; ${\dot{V}O}_{2}$ *n* = 6 vs. 10	No reliable benefit estimate because of baseline imbalance and missing intervention data	Very low	Evidence is insufficient to determine whether this mobility dose preserves ${\dot{V}O}_{2}$.
Recovery	Three programmes with different follow-up and rehabilitation exposures	Residual deficits at 5–14 d in some cohorts; one small HDBR trajectory approached baseline at ~4 weeks	Very low	Recovery timing and completeness are very uncertain and cannot be generalised.

Notes: GRADE certainty applies to the stated finding. Low certainty indicates limited confidence; very low certainty indicates very little confidence in the magnitude or inference. CI, confidence interval; CRF, cardiorespiratory fitness; HBR, horizontal bed rest; HDBR, head-down tilt bed rest; R, recovery; SD, standard deviation.

## Data Availability

All derived review data and materials will be deposited in a public repository and released with a persistent identifier upon acceptance. The complete anonymised dataset is available to the editors and reviewers on request. No individual participant data were used.

## Supplementary Materials

The following supporting information can be downloaded at: https://www.mdpi.com/article/10.3390/healthcare14203459/s1. File S1: Consolidated Supplementary Data; File S2: PRISMA 2020 Complete Checklists.

## Abbreviations

The following abbreviations are used in this manuscript:

CRF	Cardiorespiratory fitness
CPET	Cardiopulmonary exercise testing
GRADE	Grading of Recommendations Assessment, Development and Evaluation
HBR	Horizontal bed rest
HDBR	Head-down tilt bed rest
ICTRP	International Clinical Trials Registry Platform
JBI	Joanna Briggs Institute
PRISMA	Preferred Reporting Items for Systematic Reviews and Meta-Analyses
RoB 2	Cochrane Risk of Bias 2 tool
SWiM	Synthesis Without Meta-analysis
${\dot{V}O}_{2}$	Rate of oxygen uptake
