# Supplementary material for: Cardiorespiratory Fitness Decline After Short-Term Experimental Bed Rest in Older Adults and Ageing-Focused Cohorts Aged 55–65 Years: A Systematic Review
Source: Healthcare (Basel). 2026 Oct 15;14(20):3459. doi: 10.3390/healthcare14203459 (PMC13704191; doi:10.3390/healthcare14203459)
Supplement: Supplementary file 1 [file healthcare-14-03459-s001.zip › File S1 Consolidated Supplementary Data.pdf]

Supplementary Materials

Cardiorespiratory Fitness Decline after Short-Term Experimental Bed Rest in Older Adults and Ageing-Focused Cohorts Aged 55–65 Years: A Systematic Review

File S1. Consolidated Supplementary Data

Contents

1. Supplementary S1. Search sources, yields, and complete strategies (PRISMA-S [8])
2. Supplementary S2. Study selection, reports not retrieved, and full-text exclusions
3. Supplementary S3. Report-to-cohort/protocol map and dependency
4. Supplementary S4. Detailed cohort, bed-rest, CPET, and operational definitions
5. Supplementary S5. Structured quantitative outcome data, countermeasures, and recovery
6. Supplementary S6. Outcome-level risk of bias and assessment of missing results (RoB 2 [11]; JBI [12])
7. Supplementary S7. GRADE certainty [13] and SWiM compliance [10]
8. Supplementary S8. Protocol implementation and registry reconciliation
9. Supplementary S9. Data provenance, software, availability, and version control
10. Supplementary S10. Supplementary figures

Supplementary S1. Search sources, yields, and complete strategies (PRISMA-S [8])

Bibliographic databases were searched from inception to 24 May 2026, except Embase and SPORTDiscus, which were searched to 26 May 2026. ClinicalTrials.gov and WHO ICTRP were searched on 24 May 2026. No language or publication-date restrictions were applied.

Source	Search component	Records	Search date	Use
PubMed/MEDLINE	Main bibliographic search	383	24 May 2026	Included in bibliographic deduplication
PubMed/MEDLINE	Age-enriched recall search	92	24 May 2026	Included and labelled separately to assess unique recall
Embase	Main bibliographic search	682	26 May 2026	Included
Scopus	Main bibliographic search	346	24 May 2026	Included
Web of Science Core Collection	Main bibliographic search	284	24 May 2026	Included
Cochrane CENTRAL	Main bibliographic search	21	24 May 2026	Included
SPORTDiscus	Main bibliographic search	88	26 May 2026	Included
CINAHL	Main bibliographic search	68	24 May 2026	Included
ClinicalTrials.gov	Registry mapping and linked-report search	Not combined with bibliographic total	24 May 2026	Handled separately for registrations and missing-results assessment
WHO ICTRP	Registry mapping and linked-report search	Not combined with bibliographic total	24 May 2026	Handled separately for registrations and missing-results assessment
Google Scholar and backward/forward citation searching	Supplementary citation and companion-report searching	0 unique screening records	24 May 2026	Used for report linkage, cohort mapping, and verification; not included in the 1,964 bibliographic total
Total	Bibliographic records before deduplication	1,964	24–26 May 2026	Registry records handled separately

Supplementary S1A. Complete database and registry strategies

PubMed/MEDLINE main search

("Bed Rest"[Mesh] OR "bed rest"[tiab] OR bedrest[tiab] OR "bed-rest"[tiab] OR "strict bed rest"[tiab] OR "strict bedrest"[tiab] OR "horizontal bed rest"[tiab] OR "head-down tilt"[tiab] OR "head down tilt"[tiab] OR "head-down-tilt"[tiab] OR "head-down tilt bed rest"[tiab] OR "head down tilt bed rest"[tiab] OR "head-down bed rest"[tiab] OR "head down bed rest"[tiab] OR "head-down bedrest"[tiab] OR HDBR[tiab] OR "antiorthostatic bed rest"[tiab] OR "anti-orthostatic bed rest"[tiab])
AND
("Oxygen Consumption"[Mesh] OR "Exercise Test"[Mesh] OR VO2max[tiab] OR "VO2 max"[tiab] OR "V O2 max"[tiab] OR VO2peak[tiab] OR "VO2 peak"[tiab] OR "V O2 peak"[tiab] OR "peak oxygen uptake"[tiab] OR "maximal oxygen uptake"[tiab] OR "maximum oxygen uptake"[tiab] OR "maximal oxygen consumption"[tiab] OR "maximum oxygen consumption"[tiab] OR "aerobic capacity"[tiab] OR "aerobic fitness"[tiab] OR "cardiorespiratory fitness"[tiab] OR "cardio-respiratory fitness"[tiab] OR "exercise capacity"[tiab] OR "exercise tolerance"[tiab] OR "cardiopulmonary exercise test"[tiab] OR "cardiopulmonary exercise testing"[tiab] OR CPET[tiab] OR "peak aerobic power"[tiab] OR "maximal aerobic capacity"[tiab] OR "maximal aerobic power"[tiab])
NOT (animals[mh] NOT humans[mh])

PubMed/MEDLINE age-enriched recall search

("Bed Rest"[Mesh] OR "bed rest"[tiab] OR bedrest[tiab] OR "bed-rest"[tiab] OR "head-down tilt"[tiab] OR "head down tilt"[tiab] OR "head-down tilt bed rest"[tiab] OR "head down tilt bed rest"[tiab] OR HDBR[tiab])

AND
(older[tiab] OR elderly[tiab] OR aged[tiab] OR ageing[tiab] OR aging[tiab] OR geriatric[tiab] OR "older adult"[tiab] OR "older adults"[tiab] OR "middle aged"[Mesh] OR "Aged"[Mesh] OR "55"[tiab] OR "60"[tiab] OR "65"[tiab])
AND
(VO2max[tiab] OR "VO2 max"[tiab] OR VO2peak[tiab] OR "VO2 peak"[tiab] OR "peak oxygen uptake"[tiab] OR "maximal oxygen uptake"[tiab] OR "aerobic capacity"[tiab] OR "cardiorespiratory fitness"[tiab] OR "exercise capacity"[tiab])
NOT (animals[mh] NOT humans[mh])

Embase.com main search

('bed rest'/exp OR 'bed rest':ti,ab,kw OR bedrest:ti,ab,kw OR 'bed-rest':ti,ab,kw OR 'strict bed rest':ti,ab,kw OR 'strict bedrest':ti,ab,kw OR 'horizontal bed rest':ti,ab,kw OR 'head down tilt'/exp OR 'head-down tilt':ti,ab,kw OR 'head down tilt':ti,ab,kw OR 'head-down-tilt':ti,ab,kw OR 'head-down tilt bed rest':ti,ab,kw OR 'head down tilt bed rest':ti,ab,kw OR 'head-down bed rest':ti,ab,kw OR 'head down bed rest':ti,ab,kw OR 'head-down bedrest':ti,ab,kw OR hdbr:ti,ab,kw OR 'antiorthostatic bed rest':ti,ab,kw OR 'anti-orthostatic bed rest':ti,ab,kw)
AND
('oxygen consumption'/exp OR 'exercise test'/exp OR vo2max:ti,ab,kw OR 'vo2 max':ti,ab,kw OR 'v o2 max':ti,ab,kw OR vo2peak:ti,ab,kw OR 'vo2 peak':ti,ab,kw OR 'v o2 peak':ti,ab,kw OR 'peak oxygen uptake':ti,ab,kw OR 'maximal oxygen uptake':ti,ab,kw OR 'maximum oxygen uptake':ti,ab,kw OR 'maximal oxygen consumption':ti,ab,kw OR 'maximum oxygen consumption':ti,ab,kw OR 'aerobic capacity':ti,ab,kw OR 'aerobic fitness':ti,ab,kw OR 'cardiorespiratory fitness':ti,ab,kw OR 'cardio-respiratory fitness':ti,ab,kw OR 'exercise capacity':ti,ab,kw OR 'exercise tolerance':ti,ab,kw OR 'cardiopulmonary exercise test':ti,ab,kw OR 'cardiopulmonary exercise testing':ti,ab,kw OR cpet:ti,ab,kw OR 'peak aerobic power':ti,ab,kw OR 'maximal aerobic capacity':ti,ab,kw OR 'maximal aerobic power':ti,ab,kw)
AND [humans]/lim

Scopus main search

TITLE-ABS-KEY("bed rest" OR bedrest OR "bed-rest" OR "strict bed rest" OR "strict bedrest" OR "horizontal bed rest" OR "head-down tilt" OR "head down tilt" OR "head-down-tilt" OR "head-down tilt bed rest" OR "head down tilt bed rest" OR "head-down bed rest" OR "head down bed rest" OR "head-down bedrest" OR HDBR OR "antiorthostatic bed rest" OR "anti-orthostatic bed rest")
AND
TITLE-ABS-KEY(VO2max OR "VO2 max" OR "V O2 max" OR VO2peak OR "VO2 peak" OR "V O2 peak" OR "peak oxygen uptake" OR "maximal oxygen uptake" OR "maximum oxygen uptake" OR "maximal oxygen consumption" OR "maximum oxygen consumption" OR "aerobic capacity" OR "aerobic fitness" OR "cardiorespiratory fitness" OR "cardio-respiratory fitness" OR "exercise capacity" OR "exercise tolerance" OR "cardiopulmonary exercise test" OR "cardiopulmonary exercise testing" OR CPET OR "peak aerobic power" OR "maximal aerobic capacity" OR "maximal aerobic power")

Web of Science Core Collection main search

TS=("bed rest" OR bedrest OR "bed-rest" OR "strict bed rest" OR "strict bedrest" OR "horizontal bed rest" OR "head-down tilt" OR "head down tilt" OR "head-down-tilt" OR "head-down tilt bed rest" OR "head down tilt bed rest" OR "head-down bed rest" OR "head down bed rest" OR "head-down bedrest" OR HDBR OR "antiorthostatic bed rest" OR "anti-orthostatic bed rest")
AND
TS=(VO2max OR "VO2 max" OR "V O2 max" OR VO2peak OR "VO2 peak" OR "V O2 peak" OR "peak oxygen uptake" OR "maximal oxygen uptake" OR "maximum oxygen uptake" OR "maximal oxygen consumption" OR "maximum oxygen consumption" OR "aerobic capacity" OR "aerobic fitness" OR "cardiorespiratory fitness" OR "cardio-respiratory fitness" OR "exercise capacity" OR "exercise tolerance" OR "cardiopulmonary exercise test" OR "cardiopulmonary exercise testing" OR CPET OR "peak aerobic power" OR "maximal aerobic capacity" OR "maximal aerobic power")

Cochrane CENTRAL main search

([mh "Bed Rest"] OR "bed rest":ti,ab,kw OR bedrest:ti,ab,kw OR "bed-rest":ti,ab,kw OR "strict bed rest":ti,ab,kw OR "strict bedrest":ti,ab,kw OR "horizontal bed rest":ti,ab,kw OR "head-down tilt":ti,ab,kw OR "head down tilt":ti,ab,kw OR "head-down tilt bed rest":ti,ab,kw OR "head down tilt bed rest":ti,ab,kw OR "head-down bed rest":ti,ab,kw OR "head down bed rest":ti,ab,kw OR "head-down bedrest":ti,ab,kw OR HDBR:ti,ab,kw OR "antiorthostatic bed rest":ti,ab,kw OR "anti-orthostatic bed rest":ti,ab,kw)
AND
([mh "Oxygen Consumption"] OR [mh "Exercise Test"] OR VO2max:ti,ab,kw OR "VO2 max":ti,ab,kw OR "V O2 max":ti,ab,kw OR VO2peak:ti,ab,kw OR "VO2 peak":ti,ab,kw OR "V O2 peak":ti,ab,kw OR "peak oxygen uptake":ti,ab,kw OR "maximal oxygen uptake":ti,ab,kw OR "maximum oxygen uptake":ti,ab,kw OR "maximal oxygen consumption":ti,ab,kw OR "maximum oxygen consumption":ti,ab,kw OR "aerobic capacity":ti,ab,kw OR "aerobic fitness":ti,ab,kw OR "cardiorespiratory fitness":ti,ab,kw OR "cardio-respiratory fitness":ti,ab,kw OR "exercise capacity":ti,ab,kw OR "exercise tolerance":ti,ab,kw OR "cardiopulmonary exercise test":ti,ab,kw OR "cardiopulmonary exercise testing":ti,ab,kw OR CPET:ti,ab,kw OR "peak aerobic power":ti,ab,kw OR "maximal aerobic capacity":ti,ab,kw OR "maximal aerobic power":ti,ab,kw)

CINAHL via EBSCOhost main search

(MH "Bed Rest+" OR TI ("bed rest" OR bedrest OR "bed-rest" OR "strict bed rest" OR "horizontal bed rest" OR "head-down tilt" OR "head down tilt" OR "head-down tilt bed rest" OR "head down tilt bed rest" OR "head-down bed rest" OR HDBR OR "antiorthostatic bed rest" OR "anti-orthostatic bed rest") OR AB ("bed rest" OR bedrest OR "bed-rest" OR "strict bed rest" OR "horizontal bed rest"

OR "head-down tilt" OR "head down tilt" OR "head-down tilt bed rest" OR "head down tilt bed rest" OR "head-down bed rest" OR HDBR OR "antiorthostatic bed rest" OR "anti-orthostatic bed rest"))
AND
(MH "Oxygen Consumption+" OR MH "Exercise Tests+" OR TI (VO2max OR "VO2 max" OR VO2peak OR "VO2 peak" OR "peak oxygen uptake" OR "maximal oxygen uptake" OR "maximum oxygen uptake" OR "aerobic capacity" OR "aerobic fitness" OR "cardiorespiratory fitness" OR "exercise capacity" OR "exercise tolerance" OR "cardiopulmonary exercise test" OR "cardiopulmonary exercise testing" OR CPET OR "peak aerobic power" OR "maximal aerobic capacity") OR AB (VO2max OR "VO2 max" OR VO2peak OR "VO2 peak" OR "peak oxygen uptake" OR "maximal oxygen uptake" OR "maximum oxygen uptake" OR "aerobic capacity" OR "aerobic fitness" OR "cardiorespiratory fitness" OR "exercise capacity" OR "exercise tolerance" OR "cardiopulmonary exercise test" OR "cardiopulmonary exercise testing" OR CPET OR "peak aerobic power" OR "maximal aerobic capacity"))

SPORTDiscus via EBSCOhost main search

(TI ("bed rest" OR bedrest OR "bed-rest" OR "strict bed rest" OR "horizontal bed rest" OR "head-down tilt" OR "head down tilt" OR "head-down tilt bed rest" OR "head down tilt bed rest" OR "head-down bed rest" OR HDBR OR "antiorthostatic bed rest" OR "anti-orthostatic bed rest") OR AB ("bed rest" OR bedrest OR "bed-rest" OR "strict bed rest" OR "horizontal bed rest" OR "head-down tilt" OR "head down tilt" OR "head-down tilt bed rest" OR "head down tilt bed rest" OR "head-down bed rest" OR HDBR OR "antiorthostatic bed rest" OR "anti-orthostatic bed rest") OR SU ("bed rest" OR bedrest OR "head-down tilt" OR HDBR))
AND
(TI (VO2max OR "VO2 max" OR VO2peak OR "VO2 peak" OR "peak oxygen uptake" OR "maximal oxygen uptake" OR "maximum oxygen uptake" OR "aerobic capacity" OR "aerobic fitness" OR "cardiorespiratory fitness" OR "exercise capacity" OR "exercise tolerance" OR "cardiopulmonary exercise test" OR "cardiopulmonary exercise testing" OR CPET OR "peak aerobic power" OR "maximal aerobic capacity") OR AB (VO2max OR "VO2 max" OR VO2peak OR "VO2 peak" OR "peak oxygen uptake" OR "maximal oxygen uptake" OR "maximum oxygen uptake" OR "aerobic capacity" OR "aerobic fitness" OR "cardiorespiratory fitness" OR "exercise capacity" OR "exercise tolerance" OR "cardiopulmonary exercise test" OR "cardiopulmonary exercise testing" OR CPET OR "peak aerobic power" OR "maximal aerobic capacity") OR SU ("aerobic capacity" OR "cardiorespiratory fitness" OR "oxygen consumption" OR "exercise capacity"))

ClinicalTrials.gov registry search set

Run separately and log each query:
1. "bed rest" AND (VO2 OR VO2max OR VO2peak OR "oxygen uptake" OR "aerobic capacity" OR "cardiorespiratory fitness")
2. "head-down tilt" AND (VO2 OR VO2max OR VO2peak OR "oxygen uptake" OR "aerobic capacity" OR "cardiorespiratory fitness")
3. HDBR AND (VO2 OR VO2max OR VO2peak OR "oxygen uptake" OR "aerobic capacity" OR "cardiorespiratory fitness")
4. "bed rest" AND older AND (VO2 OR "oxygen uptake" OR "aerobic capacity")

Operational rule: use trial records for publication tracking, cohort mapping, and missing-results assessment. Do not extract unpublished numerical effects unless a protocol amendment is recorded.

WHO ICTRP registry search set

Run separately and log each query:
1. bed rest VO2
2. bed rest VO2max
3. bed rest VO2peak
4. bed rest oxygen uptake
5. bed rest aerobic capacity
6. head-down tilt VO2
7. head-down tilt oxygen uptake
8. HDBR VO2

Operational rule: export available CSV/records where possible; otherwise save screenshots/PDF printout of search results and manually log trial identifiers.

Google Scholar and citation chasing

Run targeted queries. Screen the first 100 results per query or until 50 consecutive irrelevant records appear, whichever comes first. Log query date, approximate hit count, number screened, and candidate records found.
1. "bed rest" "VO2peak" "older adults"
2. "bed rest" "VO2max" "older adults"
3. "bed rest" "aerobic capacity" "older adults"
4. "head-down tilt bed rest" "VO2peak"
5. "head-down tilt bed rest" "VO2max"
6. "bed rest" "maximal aerobic capacity" "older"

Backward citation chasing: reference lists of all included studies and key reviews.
Forward citation chasing: citing records of included studies using Scopus, Web of Science, Google Scholar, or other available citation tools.

Supplementary S1B. Search management and supplementary identification

Process element	Executed procedure
Reference management and initial deduplication	Zotero version 9.0.4
Bibliographic records imported	1,964
Records after automated/manual Zotero deduplication	1,046
Residual duplicate audit	32 high-confidence clusters; 40 redundant records removed
Final title/abstract screening set	1,006 unique records
Backward/forward citation searching	No additional unique screening records after matching (n = 0); used for companion-report linkage and cohort mapping

Backward and forward citation searching, targeted Google Scholar searching, and companion-report matching were completed on 24 May 2026 after deduplication. After matching against the 1,006-record library, no additional unique screening records were identified (n = 0). These methods therefore supported report linkage and cohort mapping rather than expansion of the screening corpus.

Supplementary S2. Study selection, reports not retrieved, and full-text exclusions

Supplementary S2A. Screening and eligibility workflow

Process element	Executed procedure
Title/abstract screening	Completed in duplicate by two reviewers using predefined criteria
Full-text eligibility	Completed in duplicate by the same two reviewers
Disagreement resolution	Resolved by a third reviewer
Inter-reviewer agreement	Cohen's $\kappa = 0.80$ for title and abstract screening

The κ statistic applies only to title and abstract screening. Full-text decisions were completed in duplicate and resolved by adjudication, but no separate full-text κ is reported.

Supplementary S2B. PRISMA counts

Stage	n
Records identified from bibliographic databases	1,964
Additional unique records from citation searching	0
Duplicate records removed	958
Unique records screened	1,006
Records excluded at title/abstract	975
Reports sought for retrieval	31
Reports not retrieved	8
Reports assessed for eligibility	23
Reports excluded after full text	8
Reports retained at any level	15
Final cohort/protocol units	6

Supplementary S2C. Title/abstract decision-reason distribution

Decision	Reason	n
Exclude	No clear title/abstract evidence of eligible older/ageing 5–14-day bed-rest $\dot{V}O_2$ outcome	172
Exclude	Wrong context: clinical care/hospital/surgery/rehabilitation, not controlled experimental bed rest	155
Exclude	Wrong outcome: no $\dot{V}O_2$peak/$\dot{V}O_2$max/aerobic capacity/cardiorespiratory fitness outcome apparent	141
Exclude	Wrong exposure: no experimental bed rest/HDBR in title/abstract	109

Decision	Reason	n
Exclude	Wrong duration: bed-rest exposure outside 5–14 days	97
Exclude	Registry-only source; handled outside bibliographic screening	80
Exclude	Non-human/animal/preclinical focus	46
Exclude	Wrong population: mean/range age below 55 or young cohort only	44
Exclude	Wrong population: young cohort only	43
Exclude	Wrong exposure/comparator: dry immersion, step reduction, immobilisation, or sedentary behaviour rather than eligible HBR/HDBR	27
Exclude	Review or non-primary synthesis; citation chasing only	13
Exclude	Review or non-primary synthesis	12
Exclude	Wrong duration: main bed-rest exposure outside 5–14 days	11
Exclude	Protocol/non-outcome report; not eligible as primary evidence	10
Exclude	Wrong outcome: physiological marker without extractable $\dot{V}O_2$peak/$\dot{V}O_2$max/aerobic fitness apparent	9
Exclude	Wrong exposure: spaceflight-only or non-bed-rest analogue	4
Exclude	Residual duplicate / duplicate abstract of included conference record	1
Exclude	Retracted/withdrawn publication	1
Include	Meets title/abstract eligibility or is a high-priority primary report: older/ageing cohort, 5–14-day experimental bed rest/HDBR, and $\dot{V}O_2$/aerobic fitness outcome	11
Maybe	Potentially eligible 5–14-day bed-rest/HDBR $\dot{V}O_2$/fitness study; age/subgroup or data extractability unclear at title/abstract	12
Maybe	Related BROA/CAIS/HDBR report or protocol; retrieve for cohort mapping/dependency, not necessarily independent $\dot{V}O_2$ effect	5
Maybe	Conference/abstract precursor from older-adult bed-rest cohort with aerobic capacity/$\dot{V}O_2$ testing; retrieve only if full article does not supersede it	2
Maybe	Related older-adult 10-day bed-rest report; no explicit $\dot{V}O_2$ outcome in abstract but useful for cohort mapping	1

Supplementary S2D. Reports not retrieved

Record	First author	Year	Title	DOI	PRISMA status
9	Maeda	2000	[Effects of 6-degree head-down bed rest on physical fitness].	No DOI reported	Report not retrieved
10	Martin	1986	Plasma viscosity elevations with simulated weightlessness.	No DOI reported	Report not retrieved
11	Convertino	1986	Effect of simulated weightlessness on exercise-induced anaerobic threshold.	No DOI reported	Report not retrieved
19	Ludwig	1987	Logistic risk model for the unique effects of inherent aerobic capacity on +Gz tolerance before and after simulated weightlessness.	No DOI reported	Report not retrieved
20	Convertino	1986	Bedrest-induced peak VO_2 reduction associated with age, gender, and aerobic capacity.	No DOI reported	Report not retrieved
21	Williams	1997	Human sympathoadrenal response to exercise and LBNP after bed rest: Effect of exercise training.	No DOI reported	Report not retrieved
30	Arentson-Lantz	2017	Whey protein protects muscle mass loss of older adults during 7 days of inactivity and accelerates recovery of aerobic fitness during rehabilitation.	10.1096/fasebj.31.1_supplement.652.20	Report not retrieved
31	Arentson-Lantz	2016	Preliminary findings of the effects of leucine supplementation in bedridden older adults.	10.1096/fasebj.30.1_supplement.1164.5	Report not retrieved

Eight of the 31 reports sought for retrieval (25.8%) could not be obtained in a form sufficient for full-text eligibility assessment. Six were publications dated 1986–2000 with no DOI reported in the retrieved bibliographic record; two were FASEB conference abstracts from 2016 and 2017. The retained retrieval archive confirms non-retrieval but does not preserve a source-by-source log that can reliably distinguish subscription failure from archival unavailability, nor does it allow verification of whether author contact was attempted for each report; no such contact is therefore claimed. Their eligibility and result direction remain unknown, and possible selection or missing-results bias was considered qualitatively without assuming its direction.

Supplementary S2E. Reports excluded after full-text assessment

First author	Year	Title	DOI	Primary reason	Detailed explanation
Loeppky	1993	Effects of prolonged head-down bed rest on physiological responses to moderate hypoxia.	No DOI reported	Wrong outcome	$\dot{V}O_2max$ was estimated from maximal workload rather than assessed using respiratory gas-exchange analysis; participant age was not reported, and HDBR was combined with moderate hypoxia.
Shibasaki	2003	Exercise throughout 6 degrees head-down tilt bed rest preserves thermoregulatory responses.	10.1152/japplphysiol.00188.2003	Wrong population	Young-adult cohort; mean age below eligibility threshold. The primary $\dot{V}O_2$ assessment also followed an 18-day exposure.
Capelli	2006	Factors determining the time course of VO_2max decay during bedrest: implications for VO_2max limitation.	10.1007/s00421-006-0252-3	Wrong population	Young-adult male cohorts; no eligible older subgroup.
Sawin	1979	Exercise response to simulated weightlessness.	10.1016/0094-5765(79)90120-6	Wrong population	Young-adult male cohorts; no eligible older subgroup.
Linnarsson	2015	Effects of an artificial gravity countermeasure on orthostatic tolerance, blood volumes and aerobic power after short-term bed rest (BR-AG1).	10.1152/japplphysiol.00061.2014	Wrong population	Mean age approximately 34 years; no eligible older subgroup.
Koska	2004	Effects of endurance training on endocrine response to physical exercise after 5 days of bed rest in healthy male subjects.	10.1196/annals.1296.070	Wrong population	Young male pilots; mean age approximately 33 years.
Cautero	2002	Oxygen uptake at the onset of step-exercise before and after short duration bed rest in humans.	No DOI reported	Wrong population	Young healthy men; mean age approximately 24.5 years.
Kupraty	2025	Tilted but not down: Exercise during bed rest improves mitochondrial function in older adults.	10.1113/JP287143	Non-primary publication/commentary; no original data	Journal Club/commentary discussing Dulac 2025; no original participant data.

Supplementary S3. Report-to-cohort/protocol map and dependency

Supplementary S3A. Retained reports and roles

Report	Cohort/protocol unit	Author	Year	Short title	DOI	Role	Primary $\dot{V}O_2$	Independent effect	Evidence use
R01	C1	Hedge [14]	2023	Loss of cardiorespiratory fitness and recovery after HDBR	10.1152/japplphysiol.00726.2022	Cohort-defining $\dot{V}O_2$ report	Yes	One C1 cohort only	Primary HDBR $\dot{V}O_2$, exercise and recovery
R02	C1	Hajj-Boutros [15]	2023	Canadian Space Agency standard measures	10.1159/000534063	Associated standard-measures report	No	No	Body composition, strength, protocol context
R03	C1	Hedge [16]	2022	BROA exercise-countermeasure rationale	10.3389/fphys.2022.928313	Associated rationale/protocol report	No	No	Intervention rationale
R04	C1	Hajj-Boutros [17]	2024	Canadian Bedrest Study protocol	10.1113/EP091473	Associated protocol report	No	No	Design, schedule, intervention, recovery
R05	C1	Rabineau [18]	2025	HRV and ventilatory-threshold deconditioning	10.1152/japplphysiol.00017.2025	Associated secondary outcome report	No	No	HRV and threshold outcomes
R06	C1	Zou [19]	2023	Strength and cognitive function after HDBR	10.1002/alz.078376	Associated conference abstract	No	No	Cognitive secondary context
R07	C1	Dulac [20]	2025	Muscle and mitochondrial health after HDBR	10.1113/JP285897	Associated mechanistic report	No	No	Muscle/mitochondrial outcomes
R08	C2	Arentson-Lantz [21]	2020	Low-volume leucine supplementation	10.1152/japplphysiol.00847.2019	Cohort-defining control/countermeasure report	Yes	One C2 unit only	Primary 7-day HBR control plus leucine and recovery
R09	C2	Arentson-Lantz [22]	2019	2000 steps/day during bed rest	10.1123/japa.2018-0093	Associated countermeasure report	No	No separate control	STEP countermeasure
R10	C2	Arentson-Lantz [23]	2019	Improving dietary protein quality	10.1093/gerona/glz003	Associated countermeasure report	No	No separate control	WHEY countermeasure and recovery
R11	C3	Coker [24]	2015	Bed rest and aerobic fitness in older adults	10.1093/gerona/glu123	Cohort-defining $\dot{V}O_2$ report	Yes	C3 programme	Primary 10-day HBR report

Report	Cohort/protocol unit	Author	Year	Short title	DOI	Role	Primary $\dot{V}O_2$	Independent effect	Evidence use
R12	C3	Coker [25]	2014	Bed rest worsens fat and glucose metabolism	10.1093/gerona/glt100	Associated/nested metabolic report	No	No	Metabolic context and absolute $\dot{V}O_2$ triangulation
R13	C4	Kortebein [26]	2008	Functional impact of 10 days of bed rest	10.1093/gerona/63.10.1076	Cohort-defining/corroborative $\dot{V}O_2$ report	Yes	Uncertain relative to C3	10-day HBR corroborative evidence
R14	C5	Pišot [27]	2016	Older versus younger men after bed rest and recovery	10.1152/japplphysiol.00858.2015	Cohort-defining $\dot{V}O_2$ and recovery report	Yes	C5 independent	14-day HBR and recovery
R15	C6	Zuccarelli [28]	2025	Ageing and $\dot{V}O_2$ response after bed rest	10.1249/01.mss.0001156072.84865.d8	Abstract-only cohort report	No	Unresolved	Narrative evidence only

Abbreviations: CON, control; HBR, horizontal bed rest; HDBR, head-down tilt bed rest; LEU, leucine; MIXED, mixed-source protein diet; STEP, approximately 2,000 supervised steps/day; WHEY, whey-isolate-enriched diet.

Supplementary S3B. Final cohort/protocol dependency adjudications

Unit/network	Final adjudication
BROA/CAIS/MRA	One HDBR cohort; Hedge 2023 is the primary $\dot{V}O_2$ report. Six associated reports contribute protocol, mechanistic, or secondary information.
UTMB/NCT01846130	One cohort/protocol unit; Arentson-Lantz 2020 CON selected as the primary unit-level control. STEP/WHEY controls not counted independently.
Coker 2014/2015	Coker 2014 is associated/nested; Coker 2015 is the primary functional report.
Coker versus Kortebein	Independence could not be confirmed; participant totals were not summed and both were presented as corroborative programmes.
Pišot 2016	Direct percentage changes are primary; derived absolute post means are approximate.
Zuccarelli 2025	Abstract-only narrative evidence; independence unresolved; does not anchor certainty.
HBR versus HDBR	Synthesised separately; no pooled estimate.

Supplementary S4. Detailed cohort, bed-rest, CPET, and operational definitions

Supplementary S4A. Population and programme characteristics

ID	Cohort/programme	Country/site	Registration	Model	Days	Population	Total n	V̇O₂ n	Sex	Age	Health/activity	Applicability
C1	BROA/CAIS/MRA / Canadian Bedrest Study	Canada; McGill University Health Centre	NCT04964999	6° HDBR	14	Healthy adults aged 55–65 years; women postmenopausal	22	Control n = 9 percentage change; exercise n = 10; complete-case absolute n = 6 control and n = 9 exercise	11 women/11 men	59±3 years	Healthy; ≥2.5 h/week self-reported moderate-to-vigorous activity	Ageing cohort; healthy and relatively active; indirect to frail hospitalised patients
C2	UTMB/Paddon-Jones older-adult 7-day HBR programme	USA; UTMB Clinical Research Center	NCT01846130	Horizontal strict bed rest	7	Healthy adults aged 60–80 years	Multiple reports; usually 20 total per intervention study	Control n = 10; intervention n = 6–10 depending report	Mixed sex	~68 years	Healthy; modestly active; not formally trained	Healthy volunteer model; best-case diet and supervision
C3	UAMS/Coker/Wolfe/Evans 10-day older-adult HBR programme	USA; UAMS	No registration reported	Horizontal strict bed rest	10	Older adults aged 60–85 years; clinically stable	19 in Coker 2015	19	14 women/5 men	66±1 or 69.9±1.0 years as reported	Healthy/weight stable; free of major chronic disease	Older adults but healthy and closely monitored
C4	Kortebein/Evans/Paddon-Jones 10-day older-adult HBR cohort	USA; UAMS and UTMB	No registration reported	Horizontal strict bed rest	10	Healthy older adults; range 62–75 years	11 analysed overall	V̇O₂ n = 9	Men and women	67±5 years	Healthy community-dwelling older adults	Healthy older adults; direct geriatric relevance but limited clinical complexity
C5	PANGeA / Valdoltra ageing HBR cohort	Slovenia/Italy; Orthopedic Hospital Valdoltra	IR-aging 1200	Horizontal bed rest	14	Older men aged 55–65 years; younger comparator excluded from primary review	16 older men	16	Men only	59.6±3.4 years	Healthy; no major cardiometabolic or neuromuscular disease	Ageing male cohort; sex-specific and younger than conventional ≥65 threshold
C6	Zuccarelli 2025 elderly HBR subgroup	Italy/Slovenia network; conference abstract	No registration reported	Horizontal bed rest	10	Elderly men, mean age 69±3 years	10 elderly men	10	Men only	69±3 years	Healthy older men	Older male subgroup; limited reporting

Supplementary S4B. Bed-rest, nutrition, CPET, and assessment timing

Cohort	Bed-rest protocol	Diet/co-interventions	CPET protocol	$\dot{V}O_2$ definition	Immediate assessment	Recovery assessments	Data-quality notes
C1	Continuous supervised 6° HDBR; all activities in head-down position	Weight-maintaining standardised diet; exercise arm approximately 1 h/day multimodal exercise	Electrically braked cycle; 3 min at 15 W, then 15 W/min until cadence <65 rpm	Highest 20-s moving average; breath-by-breath gas exchange	R+1 or R+2	R+6 and R+4 weeks	Some values reconstructed with validated personalised $\dot{V}O_2$-work-rate models; invalid early peak tests excluded
C2	Strict 7-day HBR; no weight bearing	Isoenergetic diet; alanine, leucine, whey, or 2,000-step protocol depending report	Monark cycle ergometer with VMax Encore metabolic cart; graded test	Peak oxygen uptake in absolute and relative units	First post-BR testing day	After 5-day inpatient rehabilitation	Maximality criteria incompletely reported; review post means calculated from baseline and change
C3	Strict 10-day HBR; bedside commode only	Eucaloric controlled diet	Lode cycle ergometer with Vmax 29N; direct respiratory gas analysis	Peak $\dot{V}O_2$ pre/post	Immediately after 10 days	No primary $\dot{V}O_2$ recovery series	Table unit labelled $L\cdot min^{-1}$ despite values consistent with $mL\cdot kg^{-1}\cdot min^{-1}$; analytic unit adjudicated
C4	Continuous 10-day HBR with brief bathroom privileges	Eucaloric diet; 0.8 g protein/kg/day	Electrically braked cycle; sex-specific warm-up, +20 W stages, then +20 W/min to volitional fatigue/ACSM termination	$\dot{V}O_2max$ assessed using respiratory gas-exchange analysis	After completion of bed rest	No primary $\dot{V}O_2$ recovery series	$\dot{V}O_2$ outcome introduced after two participants completed protocol; analytic $\dot{V}O_2$ cohort had complete pre/post data
C5	Controlled 14-day HBR	Eucaloric Mediterranean-style diet; some components differed by subgroup	Monark 839E; 80 W warm-up, then +20 W/min to volitional exhaustion	Peak $\dot{V}O_2$ normalised to body mass	BR14	R+14 after supervised multimodal rehabilitation	Direct percentage changes; absolute post values are approximate derivations
C6	10-day HBR; abstract-level description only	Insufficiently reported	Incremental exercise test	Peak pulmonary $\dot{V}O_2$	POST10	Not reported	No full post value/dispersion or complete methodological detail

Supplementary S4C. Review workflow and software

Domain	Personnel/software	Final reporting rule
Reference management/deduplication	Zotero 9.0.4	Initial duplicate detection and reference management; followed by manual residual duplicate audit.
Screening	Two reviewers plus third-reviewer adjudication	Title/abstract and full-text assessment in duplicate; Cohen's κ = 0.80 for title/abstract screening.
Data extraction/coding	Three reviewers; standardised forms	No claim of independent duplicate extraction is made in the manuscript.

Domain	Personnel/software	Final reporting rule
Risk of bias	Three reviewers; standardised design-specific judgement framework	RoB 2 for randomised comparisons; revised JBI tool for quasi-experimental/non-randomised findings; final judgements reconciled.
Quantitative processing	R 4.5.2; RStudio 2026.04.0+526; metafor 5.0-1	Statistical processing and non-pooled outputs; no meta-analysis or summary effect.
Certainty	GRADE	Seven prespecified finding-level certainty targets.

Supplementary S4D. Operational definitions and outcome hierarchy

Term	Operational definition	Analysis rule
Population pathway 1	Cohort mean age ≥60 years.	Applies at cohort level; individual ages need not all be ≥60 unless reported.
Population pathway 2	An explicitly ageing-focused experimental programme enrolling participants aged 55–65 years.	The 55–65 criterion is the enrolled age range and does not redefine all adults aged 55–59 as older adults.
Short-term bed rest	The prespecified 5–14-day exposure window used in this review.	Review-specific operational window for acute deconditioning; not a universal definition.
Directly assessed $\dot{V}O_2$	Peak or maximal oxygen uptake obtained during an exercise test with respiratory gas-exchange analysis.	Values estimated solely from workload-based predictive equations were ineligible.
Equivalent peak/maximal measure	The highest or maximal gas-exchange oxygen-uptake value from an eligible incremental peak or maximal test, regardless of whether the source used $\dot{V}O_2$peak or $\dot{V}O_2$max.	Maximality was not assumed when criteria were absent; reporting limitations informed risk of bias.
Closest valid baseline	The last technically valid pre-bed-rest assessment.	If more than one baseline existed, the assessment closest to bed-rest initiation was prioritised.
Earliest post-bed-rest assessment	The first technically valid assessment after bed rest; for HDBR, R+1 or R+2 when no in-bed maximal test was available.	Primary immediate time point selected a priori.
Recovery	The trajectory from the earliest post-bed-rest assessment to a later post-remobilisation or post-rehabilitation assessment, also interpreted relative to baseline.	Natural return to usual activity and structured rehabilitation were not assumed equivalent.
Cohort/protocol unit	A distinct participant group exposed to a specific bed-rest programme.	All linked reports from the same participants/protocol were attached to one unit; shared controls were counted once.
Primary $\dot{V}O_2$ metric	Body-mass-normalised $\dot{V}O_2$ in $mL \cdot kg^{-1} \cdot min^{-1}$.	Absolute $\dot{V}O_2$ in $L \cdot min^{-1}$ was secondary and synthesised separately.

Supplementary S5. Structured quantitative outcome data, countermeasures, and recovery

Supplementary S5A. Full $\dot{V}O_2$ numerical extraction

Row	Cohort	Report	Arm	Model	Days	Time	n	Metric	Unit	Baseline	Post/follow-up	Change	Relative change
V01	C1	Hedge 2023 [14]	Control	6° HDBR	14	R+1/2	9	Peak $\dot{V}O_2$ normalised	$mL \cdot kg^{-1} \cdot min^{-1}$	30.2±6.1 SD	NR	NR	−13.4±12.5 SD
V02	C1	Hedge 2023 [14]	Exercise	6° HDBR	14	R+1/2	10	Peak $\dot{V}O_2$ normalised	$mL \cdot kg^{-1} \cdot min^{-1}$	32.0±7.0 SD	NR	NR	−3.5±5.2 SD
V03	C1	Hedge 2023 [14]	Control	6° HDBR	14	R+1/2	6	Peak $\dot{V}O_2$ absolute	$L \cdot min^{-1}$	2.0±0.66 SD	1.79±0.58 SD	−0.21	NR
V04	C1	Hedge 2023 [14]	Control	6° HDBR	14	R+6	6	Peak $\dot{V}O_2$ absolute	$L \cdot min^{-1}$	2.0±0.66 SD	1.89±0.56 SD	−0.11	NR
V05	C1	Hedge 2023 [14]	Control	6° HDBR	14	R+4 weeks	6	Peak $\dot{V}O_2$ absolute	$L \cdot min^{-1}$	2.0±0.66 SD	2.06±0.58 SD	0.06	NR
V06	C1	Hedge 2023 [14]	Exercise	6° HDBR	14	R+1/2	9	Peak $\dot{V}O_2$ absolute	$L \cdot min^{-1}$	2.47±0.82 SD	2.39±0.81 SD	−0.08	NR
V07	C2	Arentson-Lantz 2020 [21]	CON alanine	HBR	7	Post-BR	10	Peak $\dot{V}O_2$ normalised	$mL \cdot kg^{-1} \cdot min^{-1}$	24.5±1.9 SEM	23.3	−1.2±0.9 SEM	NR
V08	C2	Arentson-Lantz 2020 [21]	LEU	HBR	7	Post-BR	10	Peak $\dot{V}O_2$ normalised	$mL \cdot kg^{-1} \cdot min^{-1}$	23.4±1.8 SEM	20.0	−3.4±1.1 SEM	NR
V09	C2	Arentson-Lantz 2020 [21]	CON alanine	HBR	7	Post-Rehab	10	Peak $\dot{V}O_2$ normalised	$mL \cdot kg^{-1} \cdot min^{-1}$	24.5±1.9 SEM	23.3	−1.2±0.9 SEM	NR
V10	C2	Arentson-Lantz 2020 [21]	LEU	HBR	7	Post-Rehab	10	Peak $\dot{V}O_2$ normalised	$mL \cdot kg^{-1} \cdot min^{-1}$	23.4±1.8 SEM	22.3	−1.1±1.0 SEM	NR
V11	C2	Arentson-Lantz 2019 [22] (STEP)	CON	HBR	7	Post-BR	10	Peak $\dot{V}O_2$ normalised	$mL \cdot kg^{-1} \cdot min^{-1}$	24.6±1.6 SEM	23.4	−1.2±1.0 SEM	NR
V12	C2	Arentson-Lantz 2019 [22] (STEP)	STEP	HBR	7	Post-BR	6	Peak $\dot{V}O_2$ normalised	$mL \cdot kg^{-1} \cdot min^{-1}$	18.4±2.1 SEM	18.7	0.3±1.1 SEM	NR
V13	C2	Arentson-Lantz 2019 [23] (WHEY)	MIXED	HBR	7	Post-BR	10	Peak $\dot{V}O_2$ normalised	$mL \cdot kg^{-1} \cdot min^{-1}$	24.5±1.85 SEM	23.35	−1.15±0.95 SEM	NR

Row	Cohort	Report	Arm	Model	Days	Time	n	Metric	Unit	Baseline	Post/follow-up	Change	Relative change
V14	C2	Arentson-Lantz 2019 [23] (WHEY)	WHEY	HBR	7	Post-BR	10	Peak $\dot{V}O_2$ normalised	$mL \cdot kg^{-1} \cdot min^{-1}$	18.7±1.33 SEM	17.76	−0.94±1.06 SEM	NR
V15	C3	Coker 2015 [24]	Single group	HBR	10	Post-BR	19	Peak $\dot{V}O_2$ normalised	$mL \cdot kg^{-1} \cdot min^{-1}$	21.5±1.3 SEM	18.6±1.0 SEM	−2.9	−13.49
V16	C3	Coker 2014 [25]	Metabolic subset	HBR	10	Post-BR	8	Peak $\dot{V}O_2$ absolute	$L \cdot min^{-1}$	1.4±0.1 SEM	1.3±0.1 SEM	−0.1	−7.14
V17	C4	Kortebein 2008 [26]	Single group	HBR	10	Post-BR	9	$\dot{V}O_2max$ normalised	$mL \cdot kg^{-1} \cdot min^{-1}$	22.7±2.0 SEM	19.72±1.7 SEM	−2.98	−12.2±4.5 SEM
V18	C5	Pišot 2016 [27]	Older men	HBR	14	BR14	16	Peak $\dot{V}O_2$ normalised	$mL \cdot kg^{-1} \cdot min^{-1}$	26.8±4.92 SD	22.7	−4.1	−15.3±11.0 SD
V19	C5	Pišot 2016 [27]	Older men	HBR	14	R+14	16	Peak $\dot{V}O_2$ normalised	$mL \cdot kg^{-1} \cdot min^{-1}$	26.8±4.92 SD	24.31	−2.49	−9.3±13.4 SD
V20	C6	Zuccarelli 2025 [28]	Older men	HBR	10	Post-BR	10	Peak pulmonary $\dot{V}O_2$ normalised	$mL \cdot kg^{-1} \cdot min^{-1}$	24.3±7.8 SD	NR	NR	−5.0

Supplementary S5B. Data provenance and dependency notes

Row	Data status	Source	Direction	Statistical result	Dependency note	Interpretive note
V01	Directly reported percent change	Results text	Decrease	$p < 0.05$ versus PRE	Primary C1 control	Substantial early loss; normalised post mean not directly tabulated.
V02	Directly reported percent change	Results text	Small decrease	Not different from PRE	Same C1 cohort	A smaller decline than control was observed in one small trial.
V03	Directly reported complete-case means	Table 2	Decrease	$p < 0.05$ versus PRE	Complete cases only	Some peak values were reconstructed using validated personalised models after device error.
V04	Directly reported complete-case means	Table 2	Persistent decrease	$p < 0.05$ versus PRE	Complete cases only	Fitness remained reduced through day 6.
V05	Directly reported complete-case means	Table 2	Approached baseline	Not different from PRE	Complete cases only	The mean approached baseline by approximately four weeks in a small complete-case sample.

Row	Data status	Source	Direction	Statistical result	Dependency note	Interpretive note
V06	Directly reported complete-case means	Table 2	Small decrease	Not different from PRE	Same C1 cohort	No statistically significant early change was reported in this small complete-case sample.
V07	Post mean calculated from reported baseline and change	Table 3	Decrease	Significant change from baseline	Selected C2 unit-level control	Direct change and SEM reported; derived post shown for readability.
V08	Post mean calculated from reported baseline and change	Table 3	Decrease	Significant change from baseline	Countermeasure arm; same C2 unit	Evidence was insufficient to determine whether leucine preserved aerobic capacity.
V09	Post mean calculated from reported baseline and change	Table 3	Incomplete numerical return	Not statistically different from baseline	Selected C2 control	Five days of rehabilitation removed statistical difference despite residual mean decrement.
V10	Post mean calculated from reported baseline and change	Table 3	No longer statistically different from baseline	Not statistically different from baseline	Same C2 unit	Evidence was insufficient to determine whether leucine altered $\dot{V}O_2$ recovery.
V11	Post mean calculated from reported baseline and change	Table 3	Small decrease	Not significant	Overlapping C2 control; not an additional cohort	Used only to describe STEP comparison.
V12	Post mean calculated from reported baseline and change	Table 3	Small increase	Not significant	Countermeasure report; same C2 unit	Evidence was insufficient to determine whether approximately 2,000 steps/day preserved aerobic capacity.
V13	Post mean calculated from reported baseline and change	Table 4	Small decrease	No significant time effect	Overlapping C2 control	No separate cohort inference.
V14	Post mean calculated from reported baseline and change	Table 4	Small decrease	No significant time effect	Countermeasure report; same C2 unit	Evidence was insufficient to determine whether a whey-enriched diet preserved aerobic capacity.
V15	Unit adjudicated; percent calculated from reported means	Table 1	Decrease	$p = 0.002$	Primary C3 report; Coker 2014 associated	Magnitude is physiologically compatible with normalised $\dot{V}O_2$, not $L \cdot min^{-1}$.
V16	Direct means; percent calculated	Table 1	Decrease	Reported as associated report	Nested/associated with C3	Used to triangulate Coker 2015 unit and provide metabolic context.

Row	Data status	Source	Direction	Statistical result	Dependency note	Interpretive note
V17	Direct means and reported percent change	Results/Table 2	Decrease	$p = 0.04$	Potential dependency with C3	Corroborative 10-day evidence; do not sum participants with C3.
V18	Post/change derived from directly reported baseline and percent change	Results/Figure 2A	Decrease	$p < 0.001$	Independent C5 cohort	Primary presentation should emphasize directly reported percent change; derived absolute values are approximate.
V19	Post/change derived from directly reported baseline and percent change	Results/Figure 2A	Incomplete recovery	$p = 0.020$ versus baseline	Same C5 cohort	Recovery remained incomplete after 14 days.
V20	Abstract-only percentage change	Conference abstract	Decrease	$p = 0.02$	Independence unresolved	Narrative evidence only; insufficient post-BR dispersion.

Supplementary S5C. Countermeasure and recovery findings

Domain	Intervention/trajectory	Comparison/time points	Quantitative result	Final RoB	Certainty	Interpretive statement
Countermeasure	Daily multimodal exercise during 14-d HDBR (~1 h/day: interval/aerobic cycling plus upper/lower-body resistance) [14]	Exercise n = 10 vs no-exercise/passive control n = 9	Source group-by-time $p = 0.002$; R+1/2: −3.5 ± 5.2% SD vs −13.4 ± 12.5% SD; review-calculated unadjusted difference 9.9 percentage points (95% CI −0.02 to 19.82; Welch t)	Some concerns	Very low	One small trial suggested attenuation of decline; evidence remains very uncertain and does not establish efficacy or a prescription.
Countermeasure	Leucine 0.06 $g \cdot kg^{-1} \cdot meal^{-1}$ during 7-d HBR [21]	Leucine n = 10 vs alanine 0.06 $g \cdot kg^{-1} \cdot meal^{-1}$ n = 10	Change −3.4 ± 1.1 vs −1.2 ± 0.9 SEM $mL \cdot kg^{-1} \cdot min^{-1}$	Some concerns	Very low	Evidence from this single comparison was insufficient to determine whether leucine preserved $\dot{V}O_2$.
Countermeasure	Approximately 2,000 supervised steps/day during 7-d HBR (2018 ± 4 steps/day; ~22 ± 1 min/day) [22]	STEP $\dot{V}O_2$ n = 6 vs no-weight-bearing control n = 10	Calculated percentage change +1.6% vs −4.9%; marked baseline imbalance and one missing intervention result	High risk	Very low	Evidence was insufficient to determine whether this mobility dose preserved $\dot{V}O_2$; the finding remains hypothesis-generating.
Countermeasure	Whey-isolate-enriched diet during 7-d HBR (~70% of dietary protein from whey) [23]	WHEY n = 10 vs MIXED plant/animal protein sources n = 10	Calculated percentage change −5.0% vs −4.7%; large baseline $\dot{V}O_2$ imbalance	High concern	Very low	Evidence from this non-randomised comparison was insufficient to determine whether a whey-enriched diet preserved $\dot{V}O_2$.

Domain	Intervention/trajectory	Comparison/time points	Quantitative result	Final RoB	Certainty	Interpretive statement
Recovery	Hedge 2023 control [14]	PRE; R+1/2; R+6; ~R+4 weeks	Complete-case absolute means: 2.00, 1.79, 1.89, and 2.06 $L \cdot min^{-1}$ (n = 6); later longitudinal models used available data	Some concerns	Very low	One small HDBR trajectory approached baseline by ~4 weeks; not a universal recovery time.
Recovery	Arentson-Lantz 2020 alanine control [21]	Post-BR and after 5-d inpatient rehabilitation	Approximately −4.9% from baseline at both displayed time points; no longer statistically different from baseline after rehabilitation	Some concerns	Very low	Residual numerical deficit despite loss of statistical significance; no natural-recovery comparator.
Recovery	Pišot 2016 older men [27]	BR14 and R+14 after structured rehabilitation	−15.3 ± 11.0% SD and −9.3 ± 13.4% SD	Some concerns	Very low	Recovery remained incomplete at 14 days; half the cohort received whey during recovery.

Supplementary S5D. Review-calculated exercise between-group contrast

The primary HDBR report provided a repeated-measures group-by-time test (p = 0.002) but did not provide a confidence interval for the earliest post-bed-rest between-group percentage-change contrast. A supplementary unadjusted contrast was calculated from the published arm-level means, SDs, and sample sizes. This cross-sectional earliest-time-point contrast is not equivalent to the source repeated-measures interaction and is not an adjusted treatment effect.

Component	Estimate/input	Uncertainty input	Result/interpretation
Exercise arm	−3.5 percentage points	5.2 SD	n = 10
Control arm	−13.4 percentage points	12.5 SD	n = 9
Difference (exercise − control)	9.9 percentage points	SE 4.479; Welch df 10.46	95% CI −0.02 to 19.82; two-sided p = 0.050
Formula	Difference = $mean_1 - mean_0$	$SE = \sqrt{(SD_1^2/n_1 + SD_0^2/n_0)}$	Welch–Satterthwaite t interval

Supplementary S6. Outcome-level risk of bias and assessment of missing results (RoB 2 [11]; JBI [12])

Supplementary S6A. Final adjudicated outcome-level risk-of-bias judgements

Finding	Domain	Cohort/report	Result	Tool	Final judgement	Category	Weight	Rationale	Certainty implication
EXP-C1	Immediate exposure	Hedge 2023 [14] control	Peak $\dot{V}O_2$ decline after 14-day HDBR	JBI 2024	Some concerns	Some concerns	Core HDBR evidence with explicit caution	Earliest peak testing had outcome-related invalid/missing tests and mixed measured/model-estimated peak values; however, the reconstruction was internally validated and submaximal indicators corroborated the direction.	Downgrade risk of bias once; additional imprecision considered separately.
EXP-C2	Immediate exposure	Arentson-Lantz 2020 [21] CON	Peak $\dot{V}O_2$ decline after 7-day HBR	JBI 2024	Some concerns	Some concerns	Core evidence	Incomplete maximality reporting, post-bed-rest timing after early remobilisation, and a data-dependent >2-SD exclusion rule.	Moderate confidence after considering imprecision.
EXP-C3	Immediate exposure	Coker 2015 [24]	Peak $\dot{V}O_2$ decline after 10-day HBR	JBI 2024	Some concerns	Some concerns	Core evidence with mandatory unit footnote	The gas-exchange-assessed CPET result had complete follow-up, but the table unit was internally inconsistent and maximality criteria were incompletely reported.	Downgrade for reporting/measurement concern; imprecision separate.
EXP-C4	Immediate exposure	Kortebein 2008 [26]	$\dot{V}O_2max$ decline after 10-day HBR	JBI 2024	Low concern	Low	Core corroborative evidence	$\dot{V}O_2$ was assessed using respiratory gas-exchange analysis with ACSM termination criteria. The two unavailable observations resulted from the outcome being introduced after those participants had completed the protocol, rather than outcome-dependent attrition; the analytic $\dot{V}O_2$ cohort was complete.	No risk-of-bias downgrade; downgrade imprecision/indirectness as applicable.

Finding	Domain	Cohort/report	Result	Tool	Final judgement	Category	Weight	Rationale	Certainty implication
EXP-C5	Immediate exposure	Pišot 2016 [27] older men	Peak $\dot{V}O_2$ decline after 14-day HBR	JBI 2024	Low concern	Low	Core ageing-cohort evidence	Complete follow-up, gas-exchange-assessed CPET, standardised timing and controlled HBR. Cognitive training during bed rest was not judged likely to alter peak $\dot{V}O_2$, and whey was delivered during recovery rather than the immediate exposure phase.	No risk-of-bias downgrade; indirectness for male 55–65-year cohort remains separate.
EXP-C6	Immediate exposure	Zuccarelli 2025 [28]	Peak $\dot{V}O_2$ decline after 10-day HBR	JBI 2024	Insufficient information	Insufficient	Narrative only	Conference abstract did not provide sufficient information on exposure fidelity, co-interventions, missing data, complete post-bed-rest values or analysis.	Cannot anchor certainty or the principal conclusion.
REC-C1	Recovery	Hedge 2023 [14] control	Trajectory through approximately four weeks	JBI 2024	Some concerns	Some concerns	Supportive recovery evidence	The complete-case descriptive table was small, but longitudinal models used all available observations. Withdrawals/illness and unmonitored activity after discharge still limit interpretation.	Downgrade risk of bias once and imprecision separately.
REC-C2	Recovery	Arentson-Lantz 2020 [21]	Five-day rehabilitation recovery	JBI 2024	Some concerns	Some concerns	Supportive	Standardised rehabilitation and complete arm-level reporting, but no natural-recovery comparator, limited maximality detail and flexible outlier handling.	Cautious wording; imprecision remains important.

Finding	Domain	Cohort/report	Result	Tool	Final judgement	Category	Weight	Rationale	Certainty implication
REC-C5	Recovery	Pišot 2016 [27] older men	Incomplete recovery at R+14	JBI 2024	Some concerns	Some concerns	Core recovery evidence	The recovery protocol was clear and follow-up complete, but whey was given to half of the older cohort during recovery and subgroups were pooled after no detected difference.	Cautious wording; indirectness and imprecision separate.
CM-C1	Countermeasures	Hedge 2023 [14]	Effect of multimodal exercise on peak $\dot{V}O_2$	RoB 2	Some concerns	Some concerns	Single-trial comparative evidence	Randomisation and intervention delivery were adequate. Missing/invalid earliest peak tests and measurement reconstruction raised concern, but the imbalance was modest, the prediction method was validated, and ventilatory-threshold and submaximal responses supported the same treatment pattern.	Downgrade risk of bias once and imprecision once.
CM-C2-LEU	Countermeasures	Arentson-Lantz 2020 [21]	Effect of leucine on peak $\dot{V}O_2$	RoB 2	Some concerns	Some concerns	Supportive	Sequence generation/concealment were not reported, maximality criteria were sparse, and >2-SD exclusions allowed analysis flexibility.	Interpret cautiously; evidence was insufficient to determine $\dot{V}O_2$ preservation.
CM-C2-STEP	Countermeasures	Arentson-Lantz 2019 [22] (STEP)	Effect of approximately 2,000 steps/day on peak $\dot{V}O_2$	RoB 2	High risk	High	Hypothesis-generating only	Sequence concealment was unreported, baseline $\dot{V}O_2$ was markedly imbalanced, one of seven intervention participants lacked $\dot{V}O_2$ data and the trial was very small.	Very low confidence.

Finding	Domain	Cohort/report	Result	Tool	Final judgement	Category	Weight	Rationale	Certainty implication
CM-C2-WHEY	Countermeasures	Arentson-Lantz 2019 [23] (WHEY)	Effect of a whey-enriched diet on peak $\dot{V}O_2$	JBI 2024	High concern	High	Narrative only	Random allocation was not clearly reported, baseline aerobic capacity differed substantially and the analysis did not clearly address the imbalance.	Very low confidence.

Supplementary S6B. Qualitative assessment of missing results and reporting bias

Method/domain	What was assessed	Interpretation
Trial-registry searching	ClinicalTrials.gov and WHO ICTRP were searched on 24 May 2026 for registrations, linked publications, ongoing or completed but unpublished studies.	No registry-only numerical effect was used as independent evidence.
Protocol/publication matching	Registrations, protocols, site, sample, arms, timing, and investigator networks were matched.	Supported cohort mapping and identification of potentially missing outcome reports.
Citation searching	Backward and forward citation searches were matched against the deduplicated library; no additional unique records were added (n = 0).	Used for report linkage, cohort mapping, and verification.
Unobtainable reports	Eight sought reports remained unobtainable in the retained retrieval archive.	Recorded separately from full-text exclusions; author contact could not be verified, and residual reporting/retrieval uncertainty remains.
Abstract-only evidence	One abstract-only report (Zuccarelli 2025) was retained narratively.	Did not anchor the primary conclusion or certainty rating.
Formal statistical tests	Funnel plots, Egger tests, and small-study-effect tests were not performed.	Too few independent cohorts for meaningful statistical assessment.

Supplementary S7. GRADE certainty [13] and SWiM compliance [10]

Supplementary S7A. Detailed GRADE judgements

Finding	Certainty target	Starting level	Risk of bias	Inconsistency	Indirectness	Imprecision	Publication bias	Other considerations	Final certainty	Conclusion
F1	Direction of the immediate effect of 7–14 days of horizontal bed rest on $\dot{V}O_2$peak/$\dot{V}O_2$max	Low — non-randomised/quasi-experimental evidence	Not serious — two immediate findings were low concern and two had some concerns	Not serious — direction was consistently negative	Not serious for the registered experimental-bed-rest question; clinical generalisability is considered separately	Not serious for the target of direction; every primary HBR unit showed decline	Undetected; the evidence base is small but searches were comprehensive	No upgrade. A possible duration gradient was not considered sufficiently secure for upgrading.	LOW (2/4)	Observed decline was consistent; low-certainty evidence suggests that short-term HBR may reduce cardiorespiratory fitness.
F2	Magnitude and possible duration–response pattern of $\dot{V}O_2$ decline during horizontal bed rest	Low — non-randomised/quasi-experimental evidence	Serious — unit ambiguity in Coker, possible Coker/Kortebein dependency and derived absolute values for Pišot	Not serious for direction, but quantitative comparability was limited	Not serious for the experimental model; applicability to frail hospitalised patients is indirect	Serious — small cohorts, wide dispersion, no common confidence interval and uncertain independence	Undetected	No dose–response upgrade because duration was confounded by cohort, sex, baseline fitness and testing differences.	VERY LOW (1/4)	A precise expected decline and a reliable duration–response relationship are not established.
F3	Immediate change in cardiorespiratory fitness after 14-day 6° head-down tilt bed rest without exercise	Low — non-randomised within-group exposure result	Serious — invalid/missing maximal tests, mixed measured/model-estimated peak values and R+1/R+2 timing	Not applicable — single cohort	Not serious for the registered HDBR ageing-cohort question	Very serious — single small cohort with wide variability and missing maximal tests	Undetected	No upgrade	VERY LOW (1/4)	HDBR was associated with early fitness loss, but the magnitude is very uncertain.
F4	Effect of daily multimodal exercise on preservation of $\dot{V}O_2$ during 14-day HDBR	High — randomised comparison	Serious — allocation concealment not documented, invalid/missing peak tests and measurement reconstruction	Not applicable — single trial	Not serious for the registered countermeasure question; wider clinical implementation remains indirect	Very serious — one small trial, wide variability and no replication	Undetected	No upgrade	VERY LOW (1/4)	One small trial suggests that daily multimodal exercise may attenuate HDBR-related decline; a specific prescription is not established.

Finding	Certainty target	Starting level	Risk of bias	Inconsistency	Indirectness	Imprecision	Publication bias	Other considerations	Final certainty	Conclusion
F5	Effect of protein-based nutritional countermeasures (leucine or whey) on $\dot{V}O_2$ preservation	High, anchored to the randomised leucine comparison	Very serious — leucine had some concerns; WHEY had high concern and major baseline imbalance	Not serious — neither comparison provided a signal that clearly contradicted the other; both were inconclusive for $\dot{V}O_2$ preservation.	Serious — interventions and participants were highly controlled and the outcome was secondary to muscle-focused aims	Very serious — small samples, wide uncertainty and non-independent programme evidence	Undetected	No upgrade	VERY LOW (1/4)	Evidence is insufficient to determine whether leucine or whey preserves $\dot{V}O_2$ during short-term bed rest.
F6	Effect of 2,000 supervised steps/day on $\dot{V}O_2$ preservation during 7-day HBR	High — randomised comparison	Very serious — high risk from baseline imbalance, unreported concealment, one missing intervention result and analysis limitations	Not applicable — single trial	Serious — the intervention was a single daily walking bout in healthy volunteers	Very serious — extremely small sample and wide uncertainty	Undetected	No upgrade	VERY LOW (1/4)	Evidence is insufficient to determine whether approximately 2,000 steps/day preserves $\dot{V}O_2$ during bed rest.
F7	Recovery of cardiorespiratory fitness after remobilisation or structured rehabilitation	Low — non-randomised recovery trajectories	Serious — all findings had some concerns, including attrition, co-interventions or lack of natural-recovery comparators	Serious — different recovery interventions, time points and outcome formats yielded different trajectories	Not serious for the experimental recovery question; hospital recovery remains indirect	Serious — small cohorts, incomplete common metrics and no replicated time point	Undetected	No upgrade	VERY LOW (1/4)	Recovery timing and completeness are very uncertain; the ~4-week trajectory derives from one small HDBR cohort.

Supplementary S7B. SWiM reporting matrix

SWiM item	Requirement	Implementation	Status	Location
1. Grouping studies for synthesis	Describe and justify groups	Separate immediate HBR, immediate HDBR, countermeasures and recovery; further order HBR by duration	COMPLETE	Main manuscript Methods — Synthesis groups
2. Standardised metric and transformation	Describe metric or transformations	Preserve direct normalised $\dot{V}O_2$ and percentage changes; use derived values only when labelled; no forced common effect size	COMPLETE	Main manuscript Methods — Data preparation

SWiM item	Requirement	Implementation	Status	Location
3. Synthesis method	Describe method used	Structured direction/magnitude synthesis, duration ordering and trial-level comparison; no vote counting by statistical significance	COMPLETE	Main manuscript Methods — Structured synthesis
4. Criteria used to prioritise results	State which results were prioritised	Primary cohort report, gas-exchange-assessed CPET, earliest post-BR, direct change, lower risk of bias, no duplicate controls	COMPLETE	Main manuscript Methods — Result hierarchy
5. Investigation of heterogeneity	Describe factors examined	Bed-rest model, duration, age, sex, baseline fitness, CPET protocol, timing, recovery intervention and data status	COMPLETE	Main manuscript Methods/Main manuscript Results
6. Certainty of evidence	Describe method	Finding-level GRADE with explicit targets for direction, magnitude, countermeasures and recovery	COMPLETE	Main manuscript Methods — Certainty
7. Data presentation methods	Describe tables/figures	Cohort table, structured quantitative table, non-pooled effect plot, dependency map and recovery trajectory	COMPLETE	Main manuscript Results/Figures
8. Reporting results	Provide finding-level summary	Seven plain-language findings with certainty labels and restricted wording	COMPLETE	Main manuscript Results and Summary of Findings
9. Limitations of synthesis	Describe limitations	No pooled estimate, sparse evidence, dependency, mixed reporting, derived/adjudicated values and no common precision measure	COMPLETE	Discussion — Limitations

Supplementary S8. Protocol implementation and registry reconciliation

Supplementary S8A. Amendment and implementation log

Amendment/decision	Reason	Effect on review
No meta-analysis undertaken	Fewer than three independent and sufficiently comparable effects were available without reliance on dependent, derived, or unit-adjudicated data.	Application of the prespecified feasibility rule; no change to population, exposure, outcome, or unit of evidence.
Exact search dates reconciled	Database searches were confirmed as 24 May 2026, except Embase and SPORTDiscus on 26 May 2026.	Documentation correction only; no change to retrieved records.
Citation-search contribution reconciled	Backward and forward citation searching added no unique records (n = 0).	PRISMA counts remain 1,964 identified and 1,006 screened.
Zotero version documented	Reference management and initial deduplication were performed in Zotero 9.0.4.	Software documentation update.
Screening workflow clarified	Title/abstract and full-text assessment were completed in duplicate by two reviewers; a third resolved discrepancies; κ = 0.80 for title/abstract screening.	Reporting clarification; no change to eligibility decisions.
Risk-of-bias tools aligned	RoB 2 used for randomised comparisons and JBI 2024 for quasi-experimental/non-randomised findings; ROBINS-I was not used.	Methods aligned with executed appraisal.
Additional outcomes field corrected	Secondary outcomes already present in the objectives were transferred to the appropriate registry field.	No new post hoc outcome introduced.
Coker 2015 unit adjudicated	Values labelled $L \cdot min^{-1}$ were physiologically consistent with $mL \cdot kg^{-1} \cdot min^{-1}$ and a related absolute report.	Visible unit-adjudication flag retained.
UTMB reports treated as one cohort/protocol unit	LEU, WHEY, and STEP reports shared protocol and potentially shared controls.	Prevents repeated control counting.
Coker 2014 treated as associated	Likely nested metabolic report within the Coker programme.	No independent effect.
Loeppky 1993 retrieval status updated	The full text was obtained after the initial retrieval log and was assessed for eligibility.	Moved from not retrieved to full-text excluded (wrong outcome); PRISMA counts updated.
Risk-of-bias adjudication updated	Five severity disagreements were resolved in a second source-first pass.	Final consensus replaced provisional judgements.
Population descriptor clarified	The distinction between mean-age ≥60 cohorts and explicitly ageing-focused programmes enrolling adults aged 55–65 required clearer reporting.	Reporting clarification only; eligibility unchanged.
Outcome/time hierarchy and recovery definition expanded	Operational definitions were expanded for gas-exchange-assessed $\dot{V}O_2$, equivalent measures, baseline, the earliest post-bed-rest time point, and recovery.	Reporting clarification only; data selection unchanged.
Cohort/protocol terminology and independence bounds clarified	Earlier unit terminology was replaced with reader-facing cohort/protocol terminology; unresolved pairs were reported as a 4–6 range of independent groups.	No change to linkage adjudications or synthesis.

Amendment/decision	Reason	Effect on review
Exercise between-group contrast added	A between-group estimate and uncertainty were calculable from the published arm-level summaries.	A transparent unadjusted percentage-point contrast was calculated from published arm summaries; no meta-analysis performed.
Table and figure presentation expanded	CPET, timing, intervention/control, provenance, sample size, and risk-of-bias information were added at the point of interpretation.	Presentation-only revision; numerical source data and final judgements unchanged.

Supplementary S8B. Registry and protocol implementation statement

The review protocol was prospectively registered in PROSPERO (CRD420261431685). The review question, population pathways, 5–14-day exposure window, eligibility criteria, and primary outcome were unchanged. Meta-analysis was conditional on at least three independent, sufficiently comparable cohort-level effects; because this condition was not met, the prespecified structured-synthesis pathway was implemented. Administrative amendments documented the final title, exact search dates, risk-of-bias tools, outcome reporting, completed synthesis, and clarified reporting terminology. None altered the cohort/protocol-level unit of evidence, eligibility decisions, or scientific conclusions.

Supplementary S9. Data provenance, software, availability, and version control

Supplementary S9A. Data-status definitions

Data-status label	Definition	Analytic rule
Directly reported	Value appears explicitly in the report, table, figure, or supplement.	Preferred for synthesis.
Calculated from reported values	Arithmetic calculation from reported means or changes.	Formula and source retained; not presented as directly reported.
Derived from percentage change	Approximate absolute post/change obtained from reported baseline and percentage change.	Descriptive only; direct percentage is primary.
Figure-derived	Value extracted from a figure using a documented method.	Must retain figure source and reviewer verification.
Unit-adjudicated	Original unit label judged internally inconsistent with the value magnitude.	Original label retained in audit trail; analytic interpretation footnoted.
Abstract-only	Only conference abstract or incomplete report available.	Narrative only; cannot anchor certainty.
Gas-exchange-assessed $\dot{V}O_2$	Peak or maximal oxygen uptake obtained during an exercise test with respiratory gas-exchange analysis.	Eligible measurement concept; values estimated solely from workload-based prediction equations were excluded.

Supplementary S9B. Key adjudications

Issue/network	Final rule
BROA/CAIS/MRA	One HDBR cohort; Hedge 2023 is the primary $\dot{V}O_2$ report. Six associated reports contribute protocol, mechanistic, or secondary information.
UTMB/NCT01846130	One cohort/protocol unit; Arentson-Lantz 2020 CON selected as the primary unit-level control. STEP/WHEY controls not counted independently.
Coker 2014/2015	Coker 2014 is associated/nested; Coker 2015 is the primary functional report.
Coker versus Kortebein	Independence could not be confirmed; participant totals were not summed and both were presented as corroborative programmes.
Pišot 2016	Direct percentage changes are primary; derived absolute post means are approximate.
Zuccarelli 2025	Abstract-only narrative evidence; independence unresolved; does not anchor certainty.
HBR versus HDBR	Synthesised separately; no pooled estimate.

Supplementary S9C. Version control and publication dataset

Version-control element	Rule
Publication dataset identifier	BRVO2_SR_PUBLICATION_DATASET_v2.0
Core evidence status	1,006 screened records; 23 full texts; 15 retained reports; six cohort/protocol units; 20 structured $\dot{V}O_2$ rows; 13 risk-of-bias findings; seven GRADE findings.

Version-control element	Rule
Documentation reconciliation	Exact dates, Zotero version, citation-search n = 0, duplicate screening and κ, cohort/protocol terminology, age pathways, $\dot{V}O_2$ definitions, non-retrieval description, intervention details, exercise contrast, manuscript–supplement callouts, PRISMA locations, and peer-review responses were reconciled.
Unit of evidence	Cohort/protocol, not publication.
Statistical synthesis	No meta-analysis; no pooled estimate or heterogeneity statistic.
Version control	Any new report, search update, numerical correction, or change in risk-of-bias or GRADE judgements requires a versioned update and repeat audit.
Data availability	Derived review materials will be deposited in a public repository and released with a persistent identifier upon acceptance; no individual participant data were used.

Supplementary S9D. Data availability

All derived review data and materials will be deposited in a public repository and released with a persistent identifier upon acceptance. The complete anonymised dataset is available to the editors and reviewers on request. No individual participant data were used.

Supplementary S10. Supplementary figures

Figure S1. Final adjudicated risk-of-bias profile

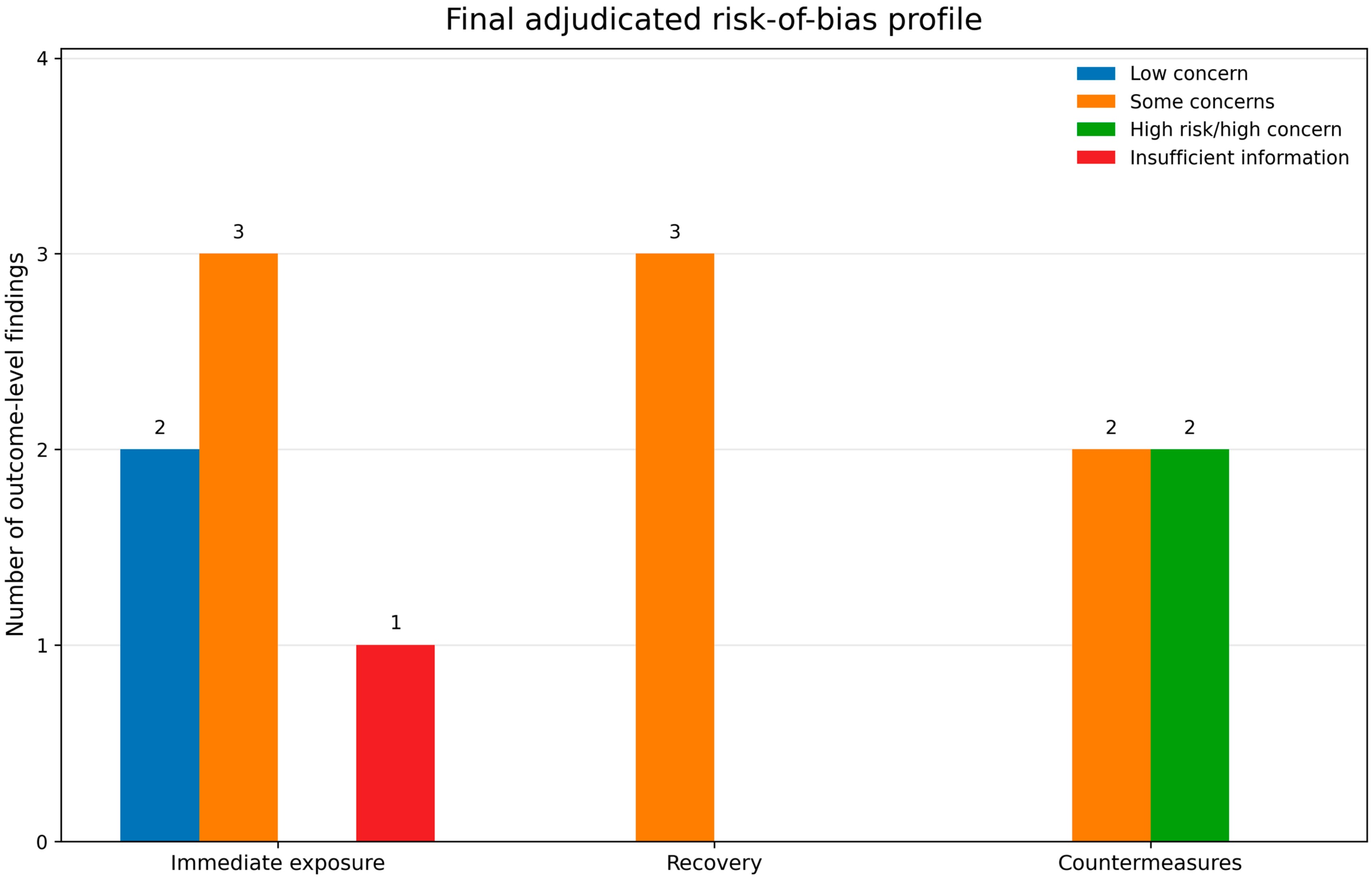

Final adjudicated outcome-level judgements for immediate exposure, recovery, and countermeasure findings. Counts refer to findings rather than publications.

Alt text: Stacked-bar chart showing two low-concern, three some-concern, and one insufficient-information immediate exposure findings; three some-concern recovery findings; and two some-concern plus two high-risk or high-concern countermeasure findings.

Figure S2. Non-pooled countermeasure changes

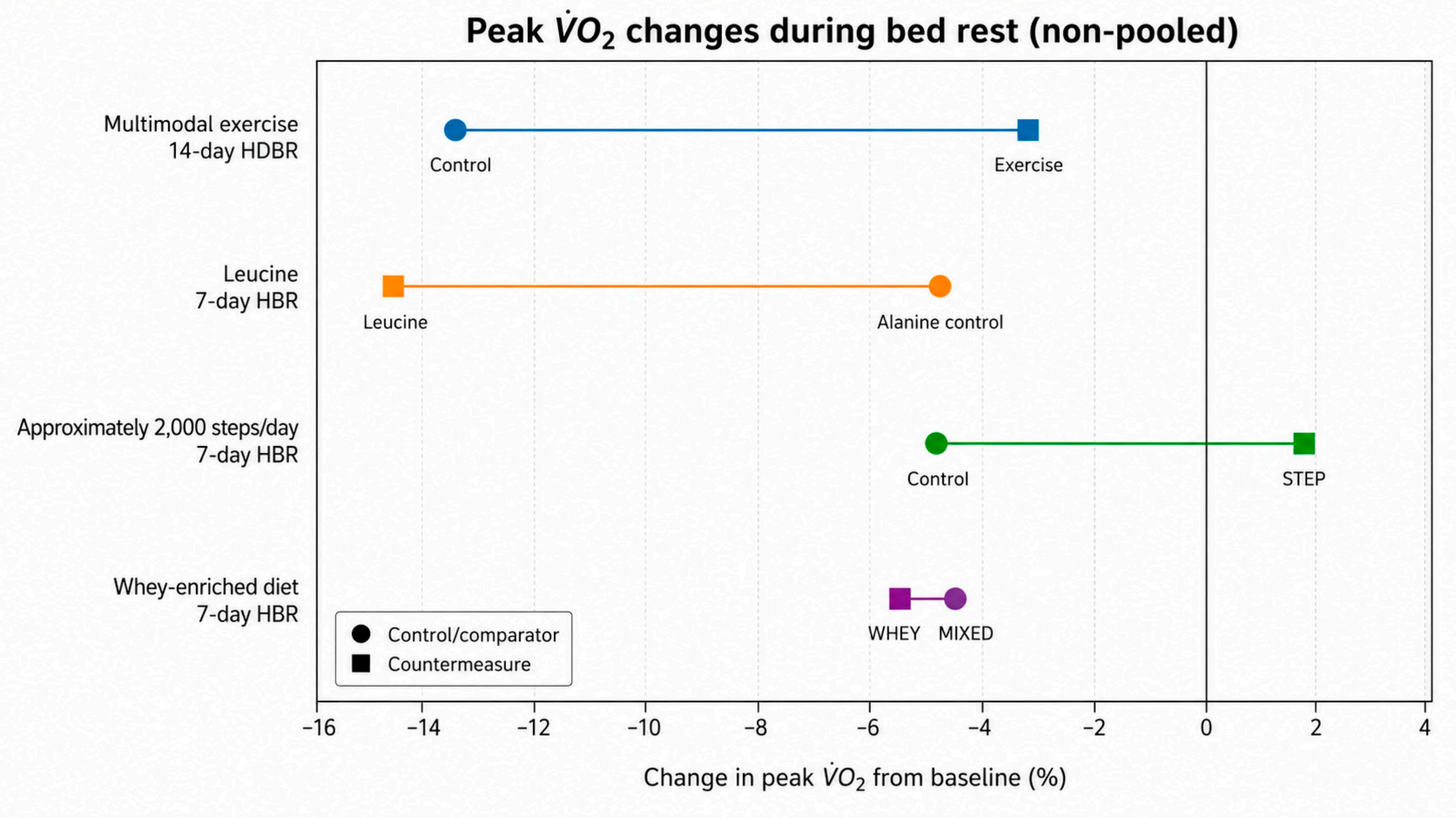

Reported or calculated arm-level percentage changes for four single countermeasure comparisons. Exercise was evaluated in one HDBR trial; leucine, STEP, and WHEY were each evaluated in one comparison within the same UTMB programme. The figure is descriptive and no pooled effect is shown. The exercise comparison arose from one small trial, and the nutritional and mobility comparisons provided insufficient evidence to determine $\dot{V}O_2$ preservation.

Alt text: Connected-point display of four non-pooled comparisons of change in peak $\dot{V}O_2$ from baseline. The exercise arm shows a smaller decline than the HDBR control in one small trial. Evidence was insufficient to determine preservation with leucine, approximately 2,000 steps/day, or a whey-enriched diet; STEP and WHEY were limited by baseline imbalance, and STEP also had missing data.
