# Supplementary material for: Cardiorespiratory Fitness Decline After Short-Term Experimental Bed Rest in Older Adults and Ageing-Focused Cohorts Aged 55–65 Years: A Systematic Review
Source: Healthcare (Basel). 2026 Oct 15;14(20):3459. doi: 10.3390/healthcare14203459 (PMC13704191; doi:10.3390/healthcare14203459)
Supplement: Supplementary file 1 [file healthcare-14-03459-s001.zip › File S2 PRISMA 2020 Complete Checklists .pdf]

File S2. PRISMA 2020 Complete Checklists

Cardiorespiratory Fitness Loss after Short-Term Experimental Bed Rest in Older Adults and Ageing Cohorts: A Systematic Review

Healthcare manuscript ID: healthcare-4518172

Contents: Part A, PRISMA 2020 main checklist (27 items and sub-items); Part B, PRISMA 2020 for Abstracts checklist (12 items). Figure 1 in the main manuscript is the PRISMA 2020 flow diagram. All locations refer to the PRISMA-reconciled manuscript v1.1 and File S1.

Part A. PRISMA 2020 Main Checklist

Section and Topic	Item #	Checklist item	Location where item is reported
**TITLE**	**TITLE**	**TITLE**	
Title	1	Identify the report as a systematic review.	Complete - Main manuscript, p. 1, title (identified as a Systematic Review).
**ABSTRACT**	**ABSTRACT**	**ABSTRACT**	
Abstract	2	See the PRISMA 2020 for Abstracts checklist.	Complete - Main manuscript, pp. 1-2, Abstract; see Part B of this file (PRISMA 2020 for Abstracts).
**INTRODUCTION**	**INTRODUCTION**	**INTRODUCTION**	
Rationale	3	Describe the rationale for the review in the context of existing knowledge.	Complete - Main manuscript, Section 1 (Introduction), p. 2.
Objectives	4	Provide an explicit statement of the objective(s) or question(s) the review addresses.	Complete - Main manuscript, final paragraph of Section 1, p. 2.
**METHODS**	**METHODS**	**METHODS**	
Eligibility criteria	5	Specify the inclusion and exclusion criteria for the review and how studies were grouped for the syntheses.	Complete - Main manuscript, Section 2.2, p. 3; File S1, Appendices 2-5 and 7.
Information sources	6	Specify all databases, registers, websites, organisations, reference lists and other sources searched or consulted to identify studies. Specify the date when each source was last searched or consulted.	Complete - Main manuscript, Section 2.2, p. 3; File S1, Appendix 1 (sources and exact search dates).
Search strategy	7	Present the full search strategies for all databases, registers and websites, including any filters and limits used.	Complete - File S1, Appendix 1A (complete database, registry, Google Scholar, and citation-search strategies).
Selection process	8	Specify the methods used to decide whether a study met the inclusion criteria of the review, including how many reviewers screened each record and each report retrieved, whether they worked independently, and if applicable, details of automation tools used in the process.	Complete - Main manuscript, Section 2.2, p. 3; File S1, Appendix 2A. Title/abstract and full-text screening were completed in duplicate by two reviewers; a third reviewer resolved discrepancies; no automation made final decisions.
Data collection process	9	Specify the methods used to collect data from reports, including how many reviewers collected data from each report, whether they worked independently, any processes for obtaining or confirming data from study investigators, and if applicable, details of automation tools used in the process.	Complete - Main manuscript, Section 2.3, p. 3; File S1, Appendices 4C and 5. Three reviewers used standardized forms; investigator contact and data-status procedures are reported. Independent duplicate extraction is not claimed.
Data items	10a	List and define all outcomes for which data were sought. Specify whether all results that were compatible with each outcome domain in each study were sought (e.g. for all measures, time points, analyses), and if not, the methods used to decide which results to collect.	Complete - Main manuscript, Sections 2.2-2.3, pp. 3-4; File S1, Appendices 4-5.
Data items	10b	List and define all other variables for which data were sought (e.g. participant and intervention characteristics, funding sources). Describe any assumptions made about any missing or unclear information.	Complete - Main manuscript, Section 2.3, p. 3; File S1, Appendices 4, 5, and 9.
Study risk of bias assessment	11	Specify the methods used to assess risk of bias in the included studies, including details of the tool(s) used, how many reviewers assessed each study and whether they worked independently, and if applicable, details of automation tools used in the process.	Complete - Main manuscript, Section 2.3, p. 3; File S1, Appendix 6. Three reviewers used design-specific tools; final judgments were reconciled. Independent duplicate appraisal is not claimed.
Effect measures	12	Specify for each outcome the effect measure(s) (e.g. risk ratio, mean difference) used in the synthesis or presentation of results.	Complete - Main manuscript, Sections 2.3 and 3.2-3.3, pp. 3-8; File S1, Appendix 5. Values are presented as baseline/post values, absolute change, and percentage change with original dispersion.
Synthesis methods	13a	Describe the processes used to decide which studies were eligible for each synthesis (e.g. tabulating the study intervention characteristics and comparing against the planned groups for each synthesis (item #5)).	Complete - Main manuscript, Section 2.3, pp. 3-4; File S1, Appendices 3 and 7B.
Synthesis methods	13b	Describe any methods required to prepare the data for presentation or synthesis, such as handling of missing summary statistics, or data conversions.	Complete - Main manuscript, Section 2.3, pp. 3-4; File S1, Appendices 5 and 9A-9B.
Synthesis methods	13c	Describe any methods used to tabulate or visually display results of individual studies and syntheses.	Complete - Main manuscript, Tables 1-2 and Figures 1-3; File S1, Appendices 5 and 10.
Synthesis methods	13d	Describe any methods used to synthesize results and provide a rationale for the choice(s). If meta-analysis was performed, describe the model(s), method(s) to identify the presence and extent of statistical heterogeneity, and software package(s) used.	Complete - Main manuscript, Section 2.3, pp. 3-4; File S1, Appendices 7B and 9. Structured synthesis without meta-analysis; R/RStudio/metafor versions are reported.
Synthesis methods	13e	Describe any methods used to explore possible causes of heterogeneity among study results (e.g. subgroup analysis, meta-regression).	Complete - Main manuscript, Sections 2.3, 3.2-3.3, and 4, pp. 3-9; File S1, Appendix 7B.

Section and Topic	Item #	Checklist item	Location where item is reported
Synthesis methods	13f	Describe any sensitivity analyses conducted to assess robustness of the synthesized results.	Not applicable / explained - No meta-analysis was undertaken and no pooled sensitivity analysis was applicable. Robustness controls (cohort dependency, source triangulation, data-status labeling, HBR/HDBR separation) are reported in Section 2.3 and File S1, Appendices 3 and 9.
Reporting bias assessment	14	Describe any methods used to assess risk of bias due to missing results in a synthesis (arising from reporting biases).	Complete - Main manuscript, Section 2.3, p. 3; File S1, Appendix 6B.
Certainty assessment	15	Describe any methods used to assess certainty (or confidence) in the body of evidence for an outcome.	Complete - Main manuscript, Section 2.3, p. 3; Table 2, p. 8; File S1, Appendix 7A.
**RESULTS**	**RESULTS**	**RESULTS**	
Study selection	16a	Describe the results of the search and selection process, from the number of records identified in the search to the number of studies included in the review, ideally using a flow diagram.	Complete - Main manuscript, Section 3.1 and Figure 1, pp. 4-5; File S1, Appendix 2B.
Study selection	16b	Cite studies that might appear to meet the inclusion criteria, but which were excluded, and explain why they were excluded.	Complete - File S1, Appendix 2E (eight excluded reports with one principal reason and detailed explanation).
Study characteristics	17	Cite each included study and present its characteristics.	Complete - Main manuscript, Section 3.1 and Table 1, pp. 5-6; File S1, Appendices 3-4.
Risk of bias in studies	18	Present assessments of risk of bias for each included study.	Complete - Main manuscript, Section 3.3, p. 8; File S1, Appendix 6A and Figure S1.
Results of individual studies	19	For all outcomes, present, for each study: (a) summary statistics for each group (where appropriate) and (b) an effect estimate and its precision (e.g. confidence/credible interval), ideally using structured tables or plots.	Complete - Main manuscript, Sections 3.2-3.3 and Figure 3, pp. 6-8; File S1, Appendix 5.
Results of syntheses	20a	For each synthesis, briefly summarise the characteristics and risk of bias among contributing studies.	Complete - Main manuscript, Sections 3.1-3.3 and Tables 1-2, pp. 4-8; File S1, Appendices 4, 6, and 7.
Results of syntheses	20b	Present results of all statistical syntheses conducted. If meta-analysis was done, present for each the summary estimate and its precision (e.g. confidence/credible interval) and measures of statistical heterogeneity. If comparing groups, describe the direction of the effect.	Complete - Main manuscript, Sections 3.2-3.3 and Figure 3, pp. 6-8; File S1, Appendices 5-7. No pooled estimate was calculated.
Results of syntheses	20c	Present results of all investigations of possible causes of heterogeneity among study results.	Complete - Main manuscript, Sections 3.2-3.3 and 4.1-4.3, pp. 6-9; File S1, Appendix 7B.
Results of syntheses	20d	Present results of all sensitivity analyses conducted to assess the robustness of the synthesized results.	Not applicable / explained - No formal pooled sensitivity analyses were conducted because no meta-analysis was performed; robustness controls are reported in Section 2.3 and File S1, Appendices 3 and 9.
Reporting biases	21	Present assessments of risk of bias due to missing results (arising from reporting biases) for each synthesis assessed.	Complete - File S1, Appendix 6B.
Certainty of evidence	22	Present assessments of certainty (or confidence) in the body of evidence for each outcome assessed.	Complete - Main manuscript, Section 3.3 and Table 2, p. 8; File S1, Appendix 7A.
**DISCUSSION**	**DISCUSSION**	**DISCUSSION**	
Discussion	23a	Provide a general interpretation of the results in the context of other evidence.	Complete - Main manuscript, Sections 4.1-4.2, pp. 8-9.
Discussion	23b	Discuss any limitations of the evidence included in the review.	Complete - Main manuscript, Section 4.3, p. 9.
Discussion	23c	Discuss any limitations of the review processes used.	Complete - Main manuscript, Section 4.3, p. 9.
Discussion	23d	Discuss implications of the results for practice, policy, and future research.	Complete - Main manuscript, Sections 4.3-4.4, p. 9.
**OTHER INFORMATION**	**OTHER INFORMATION**	**OTHER INFORMATION**	
Registration and protocol	24a	Provide registration information for the review, including register name and registration number, or state that the review was not registered.	Complete - Main manuscript, Section 2.1, p. 2: PROSPERO CRD420261431685.
Registration and protocol	24b	Indicate where the review protocol can be accessed, or state that a protocol was not prepared.	Complete - File S1, Appendix 8B. The full protocol was uploaded to the PROSPERO record (CRD420261431685); registry access/reconciliation is described there.
Registration and protocol	24c	Describe and explain any amendments to information provided at registration or in the protocol.	Complete - File S1, Appendix 8A-8B (amendment and implementation log).
Support	25	Describe sources of financial or non-financial support for the review, and the role of the funders or sponsors in the review.	Complete - Main manuscript, Funding statement, p. 10: no external funding.
Competing interests	26	Declare any competing interests of review authors.	Complete - Main manuscript, Conflicts of Interest statement, p. 10.

Section and Topic	Item #	Checklist item	Location where item is reported
Availability of data, code and other materials	27	Report which of the following are publicly available and where they can be found: template data collection forms; data extracted from included studies; data used for all analyses; analytic code; any other materials used in the review.	Complete - Main manuscript, Data Availability Statement, p. 10; File S1, Appendix 9C-9D. No individual participant data were used; derived materials will receive a persistent identifier upon acceptance.

Source: Page MJ, McKenzie JE, Bossuyt PM, et al. The PRISMA 2020 statement: an updated guideline for reporting systematic reviews. BMJ. 2021;372:n71. doi:10.1136/bmj.n71. Reproduced and completed under CC BY 4.0.

Part B. PRISMA 2020 for Abstracts Checklist

The abstract was editorially reconciled to report the PROSPERO number, principal VO_2 sample-size range, and funding statement. No numerical result, eligibility decision, risk-of-bias judgment, GRADE rating, or conclusion was changed.

Section and Topic	Item #	Checklist item	Reported (Yes/No)
**TITLE**	**TITLE**	**TITLE**	
Title	1	Identify the report as a systematic review.	Yes - Title, p. 1.
**BACKGROUND**	**BACKGROUND**	**BACKGROUND**	
Objectives	2	Provide an explicit statement of the main objective(s) or question(s) the review addresses.	Yes - Abstract, Background/Objectives, p. 1.
**METHODS**	**METHODS**	**METHODS**	
Eligibility criteria	3	Specify the inclusion and exclusion criteria for the review.	Yes - Abstract, Methods, p. 1.
Information sources	4	Specify the information sources (e.g. databases, registers) used to identify studies and the date when each was last searched.	Yes - Abstract, Methods, p. 1 (seven databases, two registries, and exact final search dates).
Risk of bias	5	Specify the methods used to assess risk of bias in the included studies.	Yes - Abstract, Methods, p. 1 (RoB 2 and revised JBI tool).
Synthesis of results	6	Specify the methods used to present and synthesise results.	Yes - Abstract, Methods, p. 1 (synthesised without meta-analysis).
**RESULTS**	**RESULTS**	**RESULTS**	
Included studies	7	Give the total number of included studies and participants and summarise relevant characteristics of studies.	Yes - Abstract, Results, pp. 1-2 (15 reports, six evidence families; principal VO_2 samples ranged from 9 to 19 participants).
Synthesis of results	8	Present results for main outcomes, preferably indicating the number of included studies and participants for each. If meta-analysis was done, report the summary estimate and confidence/credible interval. If comparing groups, indicate the direction of the effect (i.e. which group is favoured).	Yes - Abstract, Results, pp. 1-2 (HBR range, HDBR result, countermeasures, recovery, and certainty).
**DISCUSSION**	**DISCUSSION**	**DISCUSSION**	
Limitations of evidence	9	Provide a brief summary of the limitations of the evidence included in the review (e.g. study risk of bias, inconsistency and imprecision).	Yes - Abstract, Results/Conclusions, p. 2 (low/very-low certainty and uncertainty in magnitude, recovery, and countermeasure effects).
Interpretation	10	Provide a general interpretation of the results and important implications.	Yes - Abstract, Conclusions, p. 2.
**OTHER**	**OTHER**	**OTHER**	
Funding	11	Specify the primary source of funding for the review.	Yes - Abstract, final sentence, p. 2: no external funding.
Registration	12	Provide the register name and registration number.	Yes - Abstract, Methods, p. 1: PROSPERO CRD420261431685.

Source: PRISMA 2020 for Abstracts checklist, from Page MJ, McKenzie JE, Bossuyt PM, et al. BMJ. 2021;372:n71. Reproduced and completed under CC BY 4.0.
